# Supplementary material for: A specific G9a inhibitor unveils BGLT3 lncRNA as a universal mediator of chemically induced fetal globin gene expression
Source: Nat Commun. 2023 Jan 12;14:23. doi: 10.1038/s41467-022-35404-0 (PMC9837035; doi:10.1038/s41467-022-35404-0)
Supplement: Supplementary file 1 — Supplementary Information [file 41467_2022_35404_MOESM1_ESM.pdf]

A specific G9a inhibitor unveils BGLT3 lncRNA as a universal mediator of chemically induced fetal globin gene expression

Shohei Takase, Takashi Hiroyama, Fumiyuki Shirai, Yuki Maemoto, Akiko Nakata, Mayumi Arata, Seiji Matsuoka, Takeshi Sonoda, Hideaki Niwa, Shin Sato, Takashi Umehara, Mikako Shirouzu, Yosuke Nishigaya, Tatsunobu Sumiya, Noriaki Hashimoto, Ryosuke Namie, Masaya Usui, Tomokazu Ohishi, Shun-ichi Ohba, Manabu Kawada, Yoshihiro Hayashi, Hironori Harada, Tokio Yamaguchi, Yoichi Shinkai, Yukio Nakamura, Minoru Yoshida*, Akihiro Ito*

*Corresponding authors: yoshidam@riken.jp (M.Y.), aito@toyaku.ac.jp (A.I.)

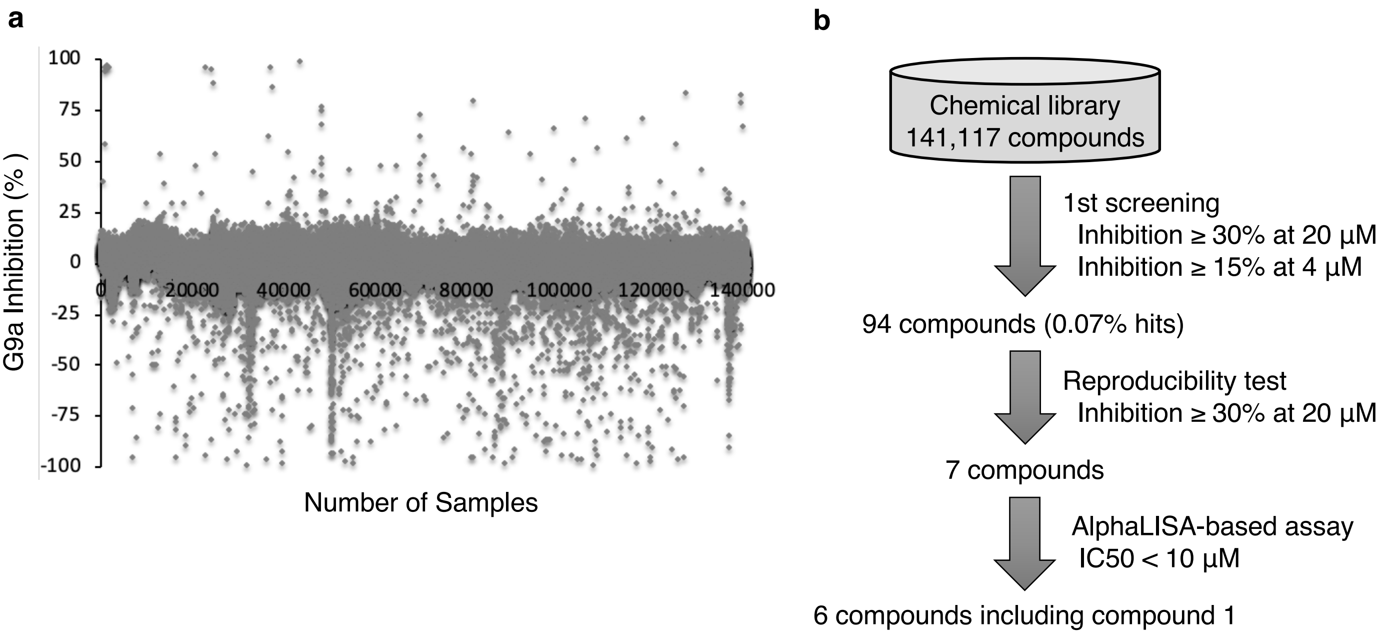

Supplementary Fig. 1. High-throughput screening for discovery of G9a inhibitors. (**a**) Scatter plot of primary screening results. (**b**) Screening flowchart to identify compound 1. Of 141,117 compounds from the DDI tested in HTS using an *in vitro* fluorogenic assay, 94 initial hit compounds were found at a hit rate of 0.07%. After testing reproducibility and calculation of IC50 values by the AlphaLISA-based assay, 6 compounds were identified as final hit compounds including compound 1.

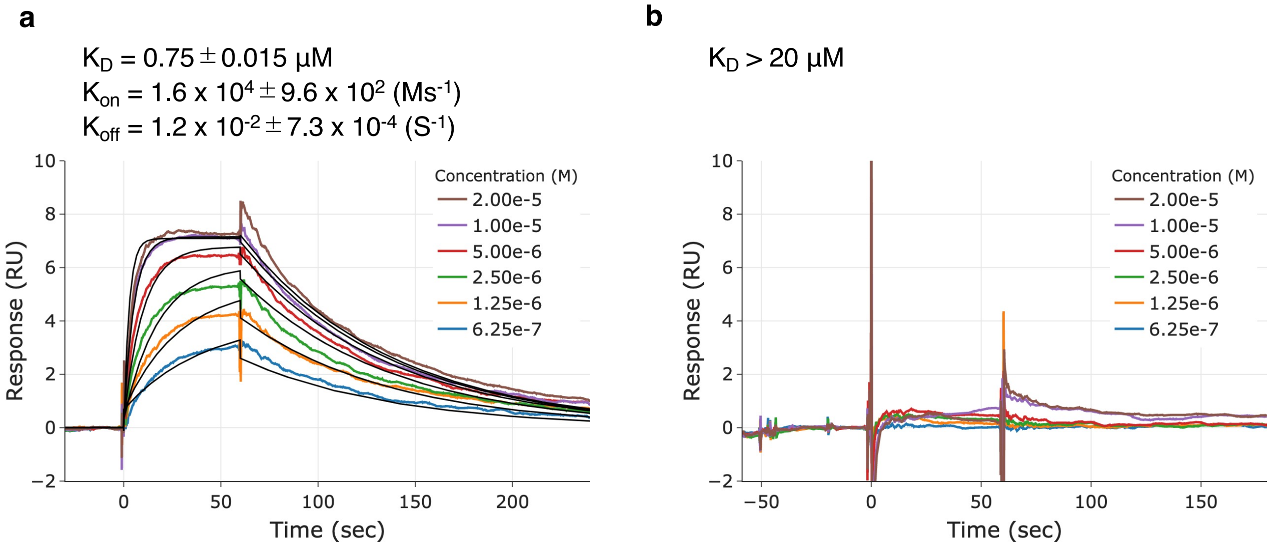

Supplementary Fig. 2. Surface plasmon resonance (SPR) analysis of binding of RK-701 and RK-0133114 to G9a.

(**a**) Kinetics analysis of RK-701 binding. Dissociation constant (K_D), association rate constant (K_{on}), and dissociation rate constant (K_{off}) shown in the figure are the mean ± SD of three kinetics runs. The sensorgram is representative from three independent runs. (**b**) SPR sensorgram of RK-0133114.

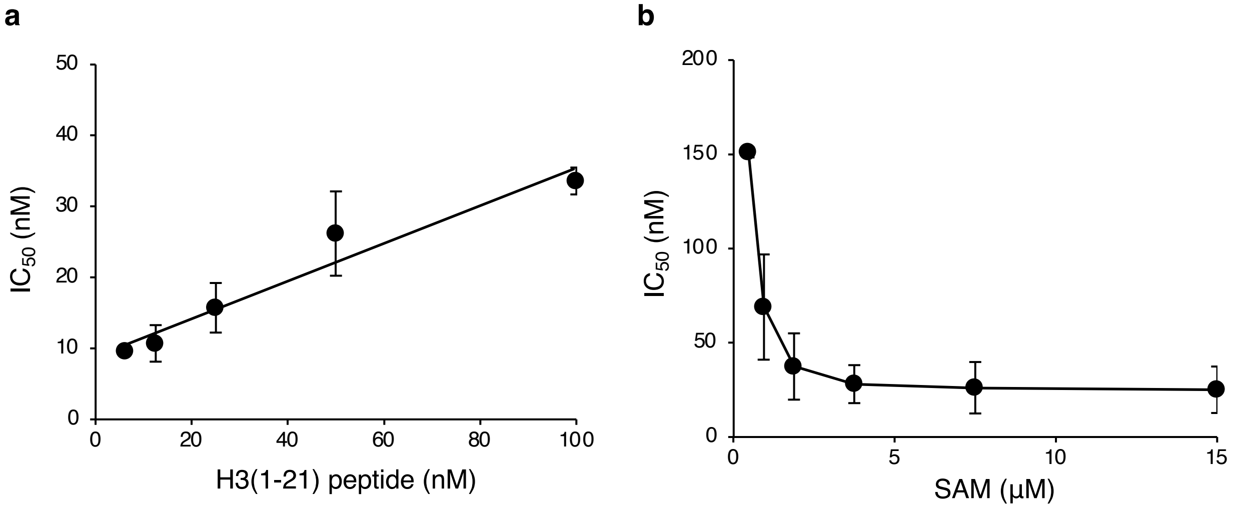

Supplementary Fig. 3. Mechanism of G9a inhibition by RK-701.

(a and **b)** IC50 vs biotinylated histone H3-derived peptide plot (a) and IC50 vs SAM plot (b) were analyzed by the AlphaLISA. Data are mean ± SD from three independent experiments.

a

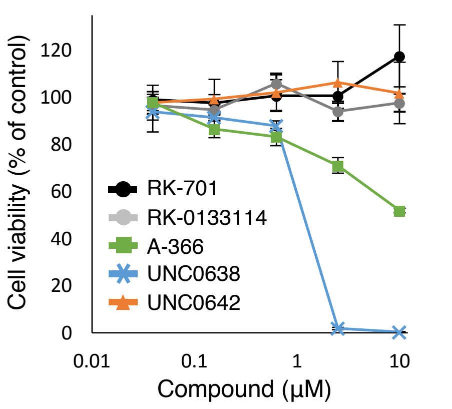

b

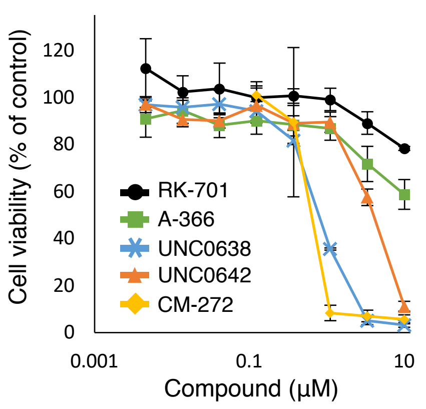

c

Dose (mg/kg)	RK-701	UNC0638	A366	UNC0642
100	3/3	N.D.	N.D.	N.D.
50	3/3	N.D.	N.D.	N.D.
25	3/3	0/3	N.D.	0/3
12.5	3/3	0/3	N.D.	1/3
6.25	N.D.	0/3	N.D.	2/3
3.125	N.D.	3/3	0/3	3/3
1.5625	N.D.	3/3	0/3	3/3
0.78125	N.D.	N.D.	3/3	N.D.
0.390625	N.D.	N.D.	3/3	N.D.
0.1953125	N.D.	N.D.	3/3	N.D.

d

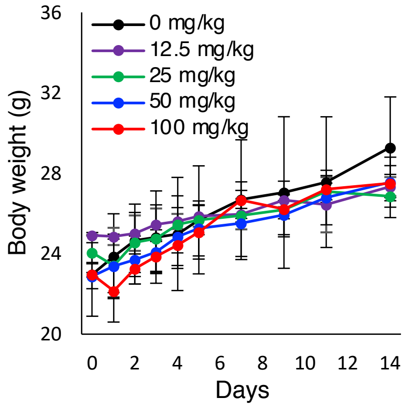

e

Compound	Concentration (µg/plate)	Number of reverants/plate			
		Base pair substitution mutation		Frameshift mutation	
		TA100		TA98	
		+ S9	- S9	+ S9	- S9
RK-701	0	121	125	36	19
	4.88	113	123	28	17
	19.5	119	105	29	13
	78.1	123	98	31	17
	313	112	118	28	13
	1250*	110	97	30	12
	2500*	104	103	28	12
	5000*	111	96	26	15
AF-2	0.01	-	727	-	-
	0.1	-	-	-	502
B[*a*]P	5.0	1375	-	288	-

f

	Dose (mg/kg)		Number of MNPCE in 4000 PCE	MNPCE (%)	No. of PCE in 500 erythrocytes	PCE (%)
RK-701	0	N	5	5	5	5
		Mean ± S.D.	6 ± 1	0.14 ± 0.04	320 ± 13	64.0 ± 2.6
		Min. / Max.	4 / 7	0.10 / 0.18	302 / 338	60.4 / 67.6
	10	N	5	5	5	5
		Mean ± S.D.	7 ± 1	0.17 ± 0.04	297 ± 21	59.5 ± 4.2
		Min. / Max.	5 / 9	0.13 / 0.23	278 / 331	55.6 / 66.2
	30	N	5	5	5	5
		Mean ± S.D.	4 ± 1	0.09 ± 0.01	307 ± 14	61.5 ± 2.8
		Min. / Max.	3 / 4	0.08 / 0.10	294 / 330	58.8 / 66.0
	100	N	5	5	5	5
		Mean ± S.D.	5 ± 1	0.13 ± 0.03	307 ± 28	61.4 ± 5.6
		Min. / Max.	4 / 7	0.10 / 0.18	258 / 328	51.6 / 65.6

Supplementary Fig. 4. Toxicities of various G9a inhibitors.

(**a**) Rat myoblast H9c2 cells were treated with the indicated G9a inhibitors for 5 days, and cell viability was evaluated by WST-8. Data are mean ± SD from three independent experiments. (**b**) HUDEP-2 cells were treated for 3 days, and cell viability was evaluated by WST-8. Data are mean ± SD from three independent experiments. (**c**) Acute toxicity of various G9a inhibitors. Number of alive mice was shown during 2-week observation. N.D. is Not Determined. (**d**) Acute toxicity of RK-701 in mice. The body weight of individual mice was measured every 1–3 days after intravenous injection. Data are presented as mean ±SD (n = 3 mice per group). (**e**) Mutagenic activity expressed as the number of revertants/plate in bacterial strains TA98 and TA100 exposed to RK-701, at various doses, with (+S9) or without (-S9) metabolic activation. Numbers represents averages from the two different experiments. AF-2 (2-(2-furyl)-3-(5-nitro-2-furyl)acrylamide) and B[a]P (benzo[a]pyrene) were used as positive controls. An asterisk indicates that insoluble material was detected. (**f**) The frequency of micronucleated polychromatic erythrocytes (MNPCE) and standard deviation of 4000 cells obtained from male rat treated with RK-701 via oral administration once a day for 2 weeks. MNCPE (%) and PCE (%) mean a proportion of MNPCE per 4000 PCE and a proportion of PCE, including MNPCE per 500 erythrocytes (n = 5 per group).

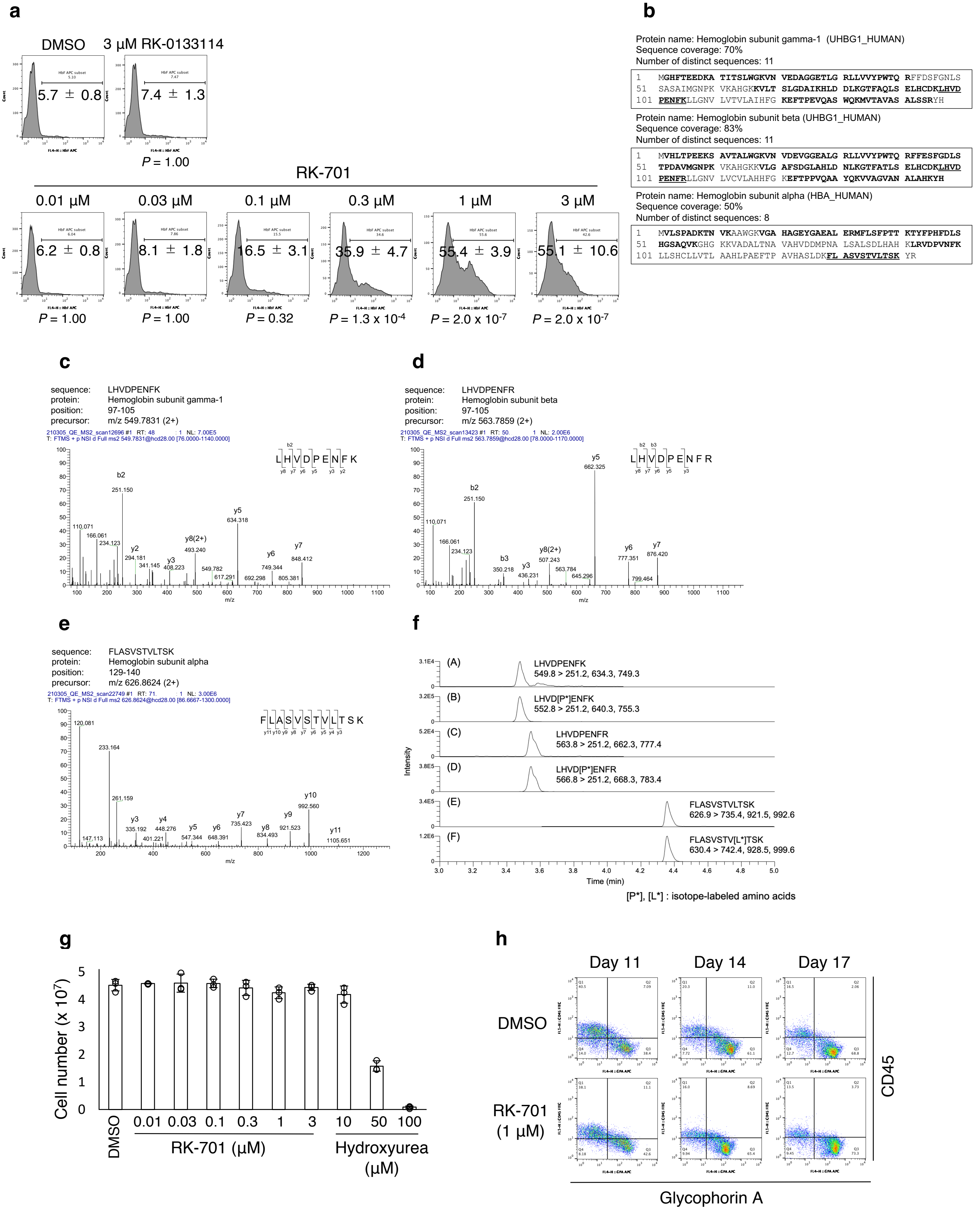

a
DMSO
3 μM RK-0133114
5.7 ± 0.8
7.4 ± 1.3
P = 1.00
RK-701
0.01 μM
0.03 μM
0.1 μM
0.3 μM
1 μM
3 μM
6.2 ± 0.8
8.1 ± 1.8
16.5 ± 3.1
35.9 ± 4.7
55.4 ± 3.9
55.1 ± 10.6
P = 1.00
P = 1.00
P = 0.32
P = 1.3 x 10-4
P = 2.0 x 10-7
P = 2.0 x 10-7
b
Protein name: Hemoglobin subunit gamma-1 (UHBG1_HUMAN)
Sequence coverage: 70%
Number of distinct sequences: 11
1 MGHFTEEDKA TITSLWGKVN VEDAGGETLG RLLVVYPWTQ RFFDSFGNLS
51 SASAIMGNPK VKAHGKKVLT SLGDAIKHLD DLKGTFAQLS ELHCDKLHVD
101 PENFKLLGNV LVTVLAIHFG KEFTPEVQAS WQKMVTAVAS ALSSRYH
Protein name: Hemoglobin subunit beta (UHBG1_HUMAN)
Sequence coverage: 83%
Number of distinct sequences: 11
1 MVHLTPEEKS AVTALWGKVN VDEVGGEALG RLLVVYPWTQ RFFESFGDLS
51 TPDAVMGNPK VKAHGKKVLG AFSDGLAHLD NLKGTFATLS ELHCDKLHVD
101 PENFRLLGNV LVCVLAHHFG KEFTPPVQAA YQKVVAGVAN ALAHKYH
Protein name: Hemoglobin subunit alpha (HBA_HUMAN)
Sequence coverage: 50%
Number of distinct sequences: 8
1 MVLSPADKTN VKAAWGKVGA HAGEYGAEAL ERMFLSFPTT KTYFPHFDLS
51 HGSAQVKGHG KKVADALTNA VAHVDDMPNA LSALSDLHAH KLRVDPVNFK
101 LLSHCLLVTL AAHLPAEFTP AVHASLDKFL ASVSTVLTSK YR
c
sequence: LHVDPENFK
protein: Hemoglobin subunit gamma-1
position: 97-105
precursor: m/z 549.7831 (2+)
210305_QE_MS2_scan12696 #1 RT: 48 1 NL: 7.00E5
T: FTMS + p NSI d Full ms2 549.7831@hcd28.00 [76.0000-1140.0000]
m/z
d
sequence: LHVDPENFR
protein: Hemoglobin subunit beta
position: 97-105
precursor: m/z 563.7859 (2+)
210305_QE_MS2_scan13423 #1 RT: 50. 1 NL: 2.00E6
T: FTMS + p NSI d Full ms2 563.7859@hcd28.00 [78.0000-1170.0000]
m/z
e
sequence: FLASVSTVLTSK
protein: Hemoglobin subunit alpha
position: 129-140
precursor: m/z 626.8624 (2+)
210305_QE_MS2_scan22749 #1 RT: 71. 1 NL: 3.00E6
T: FTMS + p NSI d Full ms2 626.8624@hcd28.00 [86.6667-1300.0000]
m/z
f
(A) LHVDPENFK 549.8 > 251.2, 634.3, 749.3
(B) LHVD[P*]ENFK 552.8 > 251.2, 640.3, 755.3
(C) LHVDPENFR 563.8 > 251.2, 662.3, 777.4
(D) LHVD[P*]ENFR 566.8 > 251.2, 668.3, 783.4
(E) FLASVSTVLTSK 626.9 > 735.4, 921.5, 992.6
(F) FLASVSTV[L*]TSK 630.4 > 742.4, 928.5, 999.6
Intensity
Time (min)
[P*], [L*] : isotope-labeled amino acids
g
Cell number (x 10^7)
DMSO
0.01 0.03 0.1 0.3 1 3
RK-701 (μM)
10 50 100
Hydroxyurea (μM)
h
Day 11
Day 14
Day 17
DMSO
RK-701 (1 μM)
CD45
Glycophorin A

Supplementary Fig. 5. Effect of G9a inhibitors and hydroxyurea on HbF induction, cell viability and differentiation of human erythroid cells.

(**a**) Flow cytometric analysis of γ-globin expression in HUDEP-2 cells treated with RK-701 or RK-0133114 for 11 days. Percentages of HbF-expressing cells were determined by gating HbF-positive cells. Data are mean ± SD from three independent experiments. (**b**) Peptides identified as part of the hemoglobin subunit by a Mascot database search are shown in bold. The underlined sequences were used as targets of MRM measurement. (**c–e**) The product ion spectrum from precursor peptides of hemoglobin subunit γ-1 (**c**), β (**d**), and α (**e**) are labeled as b- and y-type ions. (**f**) The chromatograms in (A), (C), and (E) indicate native peptides formed by trypsinization, whereas those in (B), (D), and (F) are their internal standards containing the isotope-labeled amino acid. (**g**) $CD34^+$ cells were treated with RK-701 or hydroxyurea for 10 days under conditions favoring erythroid cell differentiation. Cell viability was measured by cell counting. Data represent means ± SD from three independent experiments. (**h**) Flow cytometric analyses of erythroid cell differentiation from $CD34^+$ cells in days 11, 14, and 17. CD45 and glycophorin A were used as markers for hematopoietic cells and erythroid cells, respectively. *P*-value was calculated by one-way ANOVA with Tukey's post hoc test.

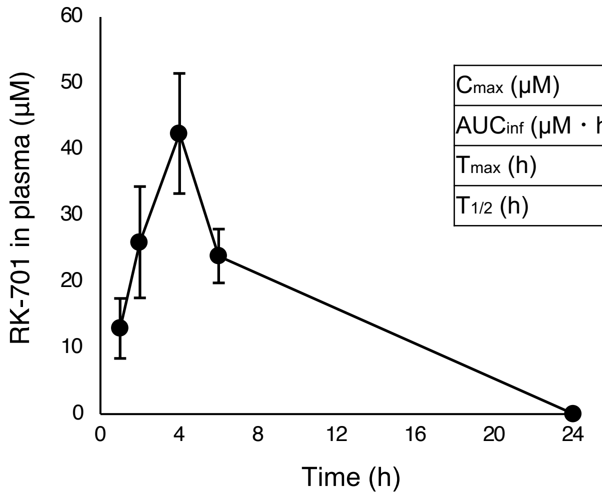

C_{max} (μM)	42.4 ± 9.1
AUC_{inf} (μM・h)	418 ± 7
T_{max} (h)	4.0 ± 0
$T_{1/2}$ (h)	1.4 ± 0.1

Supplementary Fig. 6. *In vivo* pharmacokinetic properties of RK-701 in mice.

Time course of the RK-701 concentration in plasma after a single intraperitoneal administration (25 mg/kg) was measured by LC–MS/MS. Data are means ± s.e.m. (n = 3 mice).

a

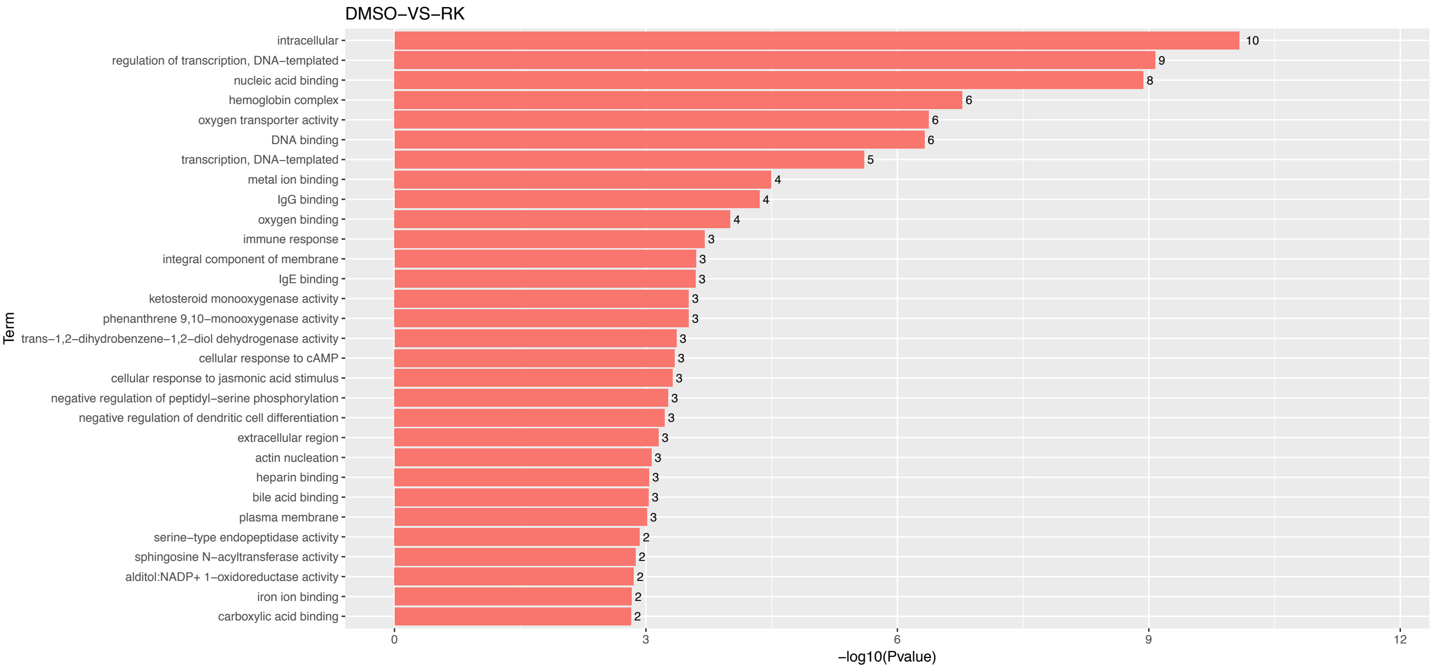

b

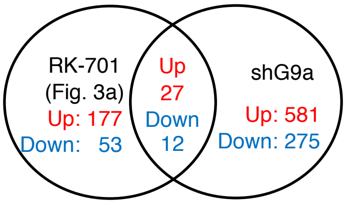

Supplementary Fig. 7. RNA-seq analyses for G9a inhibition in HUDEP-2 cells.

(**a**) Gene Ontology (GO) enrichment *p*-value histogram used gene list is Supplementary Table 6. X axis: -log10(*p*-value) of each term. Y axis: significant enriched GO term. The number differentially expressed genes in each GO term is shown in the histogram with the specification of the relevant biological process, cellular component and molecular function. Shown is the top 30 most prominent GO categories. All the output is shown if the analysis returns less than 30 outputs. The software we used here is GOSeq[1], which based on an extension of the hypergeometric distribution known as the Wallenius non- central hyper-geometric distribution. This method is able to account for gene length bias and read counts bias when performing GO analysis. Threshold for filtering here is: over represented *p*-value <= 0.05. The statistical test was performed by Wallenius in goseq software. (**b**) Venn diagram showing the overlap of differentially expressed genes between treatment with 1 μM RK-701 for 4 days and shRNA-mediated G9a knockdown in HUDEP-2 cells. Results from three biological replicates are shown.

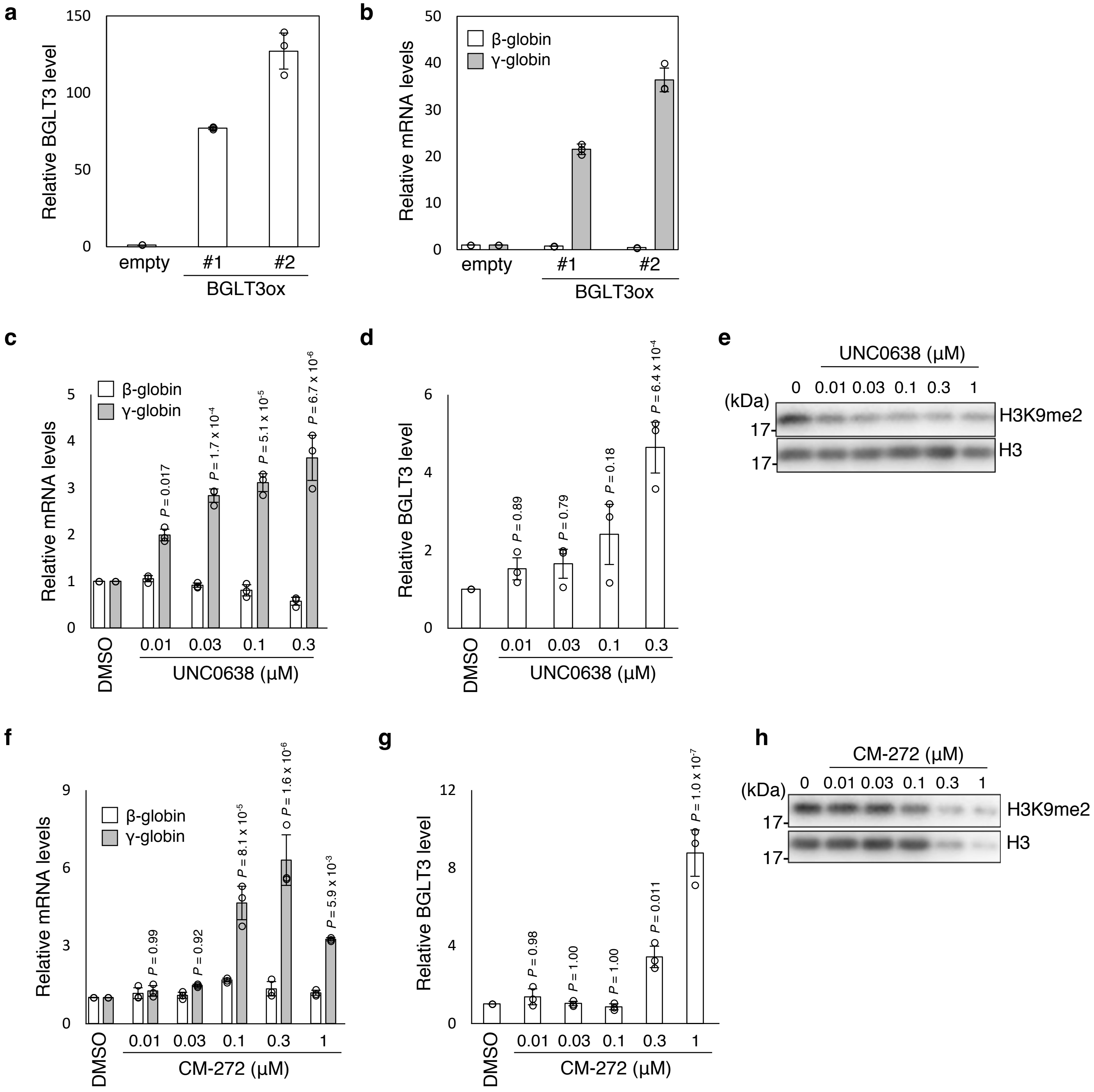

a
Relative BGLT3 level
empty
#1
#2
BGLT3ox
b
Relative mRNA levels
β-globin
γ-globin
empty
#1
#2
BGLT3ox
c
Relative mRNA levels
β-globin
γ-globin
P = 0.017
P = 1.7 x 10-4
P = 5.1 x 10-5
P = 6.7 x 10-6
DMSO
UNC0638 (μM)
d
Relative BGLT3 level
P = 0.89
P = 0.79
P = 0.18
P = 6.4 x 10-4
DMSO
UNC0638 (μM)
e
UNC0638 (μM)
(kDa)
H3K9me2
H3
f
Relative mRNA levels
β-globin
γ-globin
P = 0.99
P = 0.92
P = 8.1 x 10-5
P = 1.6 x 10-6
P = 5.9 x 10-3
DMSO
CM-272 (μM)
g
Relative BGLT3 level
P = 0.98
P = 1.00
P = 1.00
P = 0.011
P = 1.0 x 10-7
DMSO
CM-272 (μM)
h
CM-272 (μM)
(kDa)
H3K9me2
H3

Supplementary Fig. 8. Induction of fetal globin by G9a inhibition via BGLT3 in HUDEP-2 cells.

(**a**) Validation of BGLT3 overexpression by qPCR in HUDEP-2 cells. Data are mean ± SD from three independent experiments. (**b**) Relative expression of β- and γ-globin genes in BGLT3-overexpressing HUDEP-2 cells. Data are mean ± SD from three independent experiments. (**c** and **d**) Relative expression of β- and γ-globin (c) and BGLT3 (d) genes in HUDEP-2 cells treated with UNC0638 for 4 days. Data are mean ± SD from three independent experiments. (**e**) Effects of UNC0638 on H3K9me2 in HUDEP-2 cells. HUDEP-2 cells were treated with different concentrations of UNC0638 for 4 days. Lysates were immunoblotted with the indicated antibodies. A representative image of three independent experiments was shown. (**f** and **g**) Relative expression of β- and γ-globin (f) and BGLT3 (g) genes in HUDEP-2 cells treated with CM-272 for 4 days. Data are mean ± SD from three independent experiments. (**h**) Effects of CM-272 on H3K9me2 in HUDEP-2 cells. HUDEP-2 cells were treated with different concentrations of CM-272 for 4 days. Lysates were immunoblotted with the indicated antibodies. A representative image of three independent experiments was shown. *P*-value was calculated by one-way ANOVA with Tukey's post hoc test.

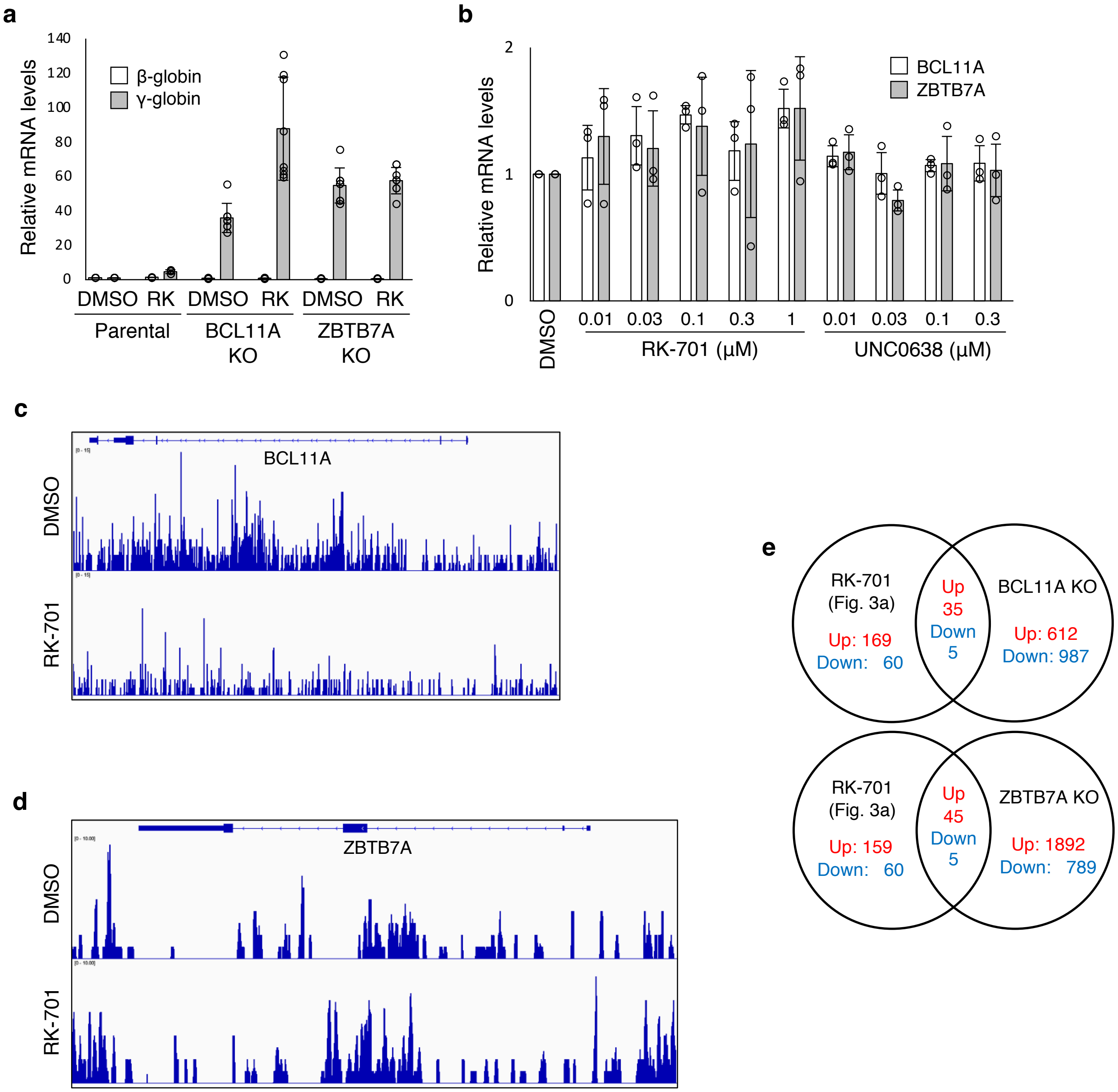
a
Relative mRNA levels
140
120
100
80
60
40
20
0
β-globin
γ-globin
DMSO RK DMSO RK DMSO RK
Parental
BCL11A KO
ZBTB7A KO
b
Relative mRNA levels
2
1
0
BCL11A
ZBTB7A
DMSO
0.01 0.03 0.1 0.3 1
RK-701 (μM)
0.01 0.03 0.1 0.3
UNC0638 (μM)
c
BCL11A
DMSO
RK-701
d
ZBTB7A
DMSO
RK-701
e
RK-701
(Fig. 3a)
Up: 169
Down: 60
Up
35
Down
5
BCL11A KO
Up: 612
Down: 987
RK-701
(Fig. 3a)
Up: 159
Down: 60
Up
45
Down
5
ZBTB7A KO
Up: 1892
Down: 789

Supplementary Fig. 9. Effect of RK-701 on gene expression of major fetal globin repressors and γ-globin, and H3K9me2 chromatin occupancy at the major fetal globin repressors locus.

(**a**) Relative expression of β- and γ-globin in parental, BCL11A-, or ZBTB7A-knockout HUDEP-2 cells treated with 1 μM RK-701 for 4 days. Data are mean ± SD from three independent experiments. (**b**) Relative expression of BCL11A and ZBTB7A in HUDEP-2 cells treated with RK-701 or UNC0638 for 4 days. Data are mean ± SD from three independent experiments. (**c** and **d**) ChIP-seq traces for H3K9me2 on the BCL11A (c) or ZBTB7A (d) gene locus in HUDEP-2 cells treated with 1 μM RK-701 or DMSO for 4 days. A representative image of two independent experiments was shown. (**e**) Venn diagram showing the overlap of differentially expressed genes between treatment with 1 μM RK-701 for 4 days in HUDEP-2 cells and BCL11A- or ZBTB7A-knockout HUDEP-2 cells. Results from three biological replicates was shown.

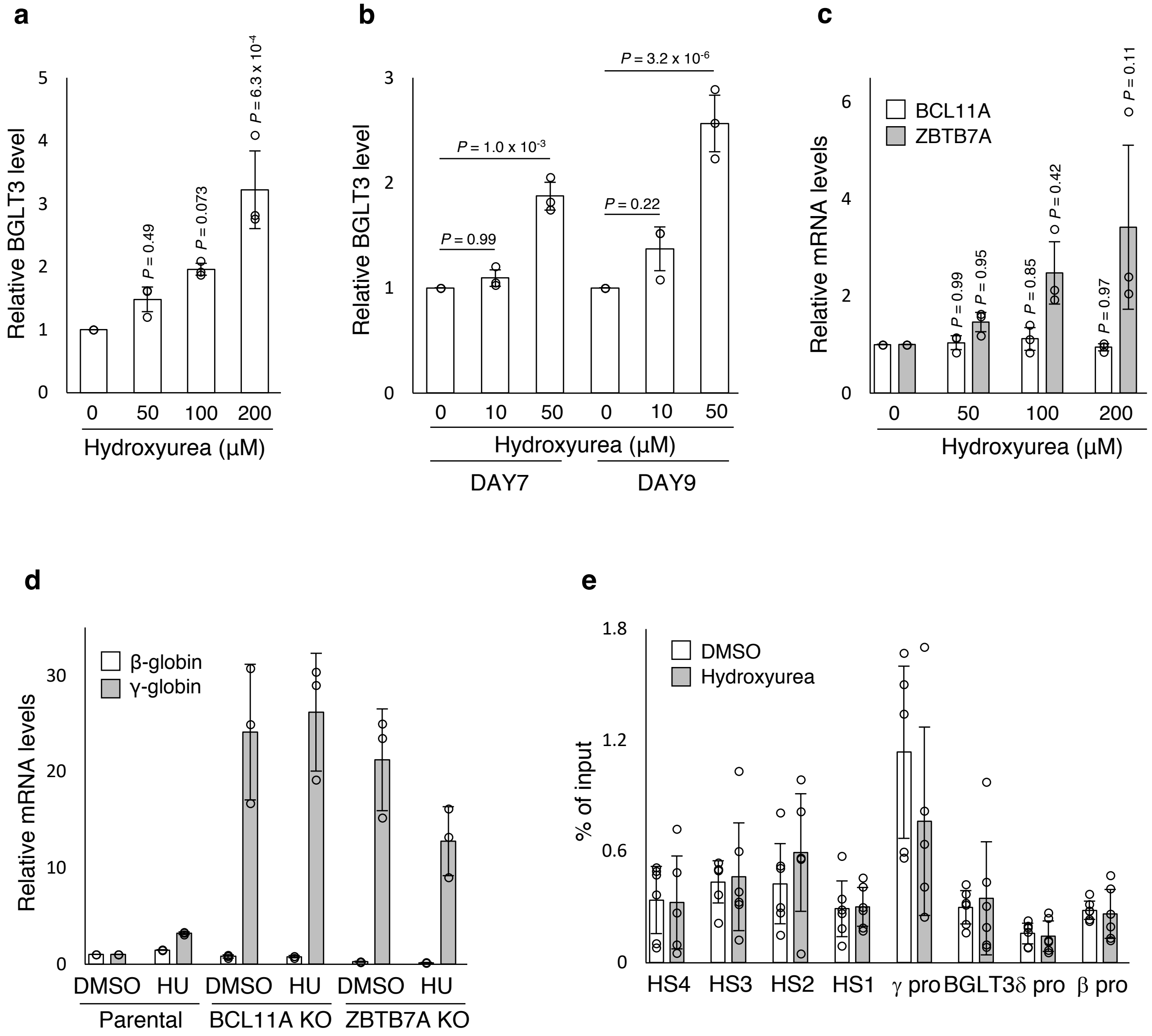
a
Relative BGLT3 level
P = 0.49
P = 0.073
P = 6.3 x 10-4
0
50
100
200
Hydroxyurea (μM)
b
Relative BGLT3 level
P = 0.99
P = 1.0 x 10-3
P = 0.22
P = 3.2 x 10-6
Hydroxyurea (μM)
DAY7
DAY9
c
Relative mRNA levels
BCL11A
ZBTB7A
P = 0.99
P = 0.95
P = 0.85
P = 0.42
P = 0.97
P = 0.11
Hydroxyurea (μM)
d
Relative mRNA levels
β-globin
γ-globin
DMSO
HU
Parental
BCL11A KO
ZBTB7A KO
e
% of input
DMSO
Hydroxyurea
HS4
HS3
HS2
HS1
γ pro
BGLT3
δ pro
β pro

Supplementary Fig. 10 Effect of hydroxyurea on BGLT3 and repressors gene expression and H3K9me2 chromatin occupancy in HUDEP-2 cells.

(**a**) Induction of BGLT3 by hydroxyurea. Relative expression of BGLT3 in HUDEP-2 cells treated with indicated concentrations of hydroxyurea for 4 days. Data are mean ± SD from three independent experiments. (**b**) Relative expression of BGLT3 in $CD34^+$ cells treated with indicated concentrations of hydroxyurea (HU) for 7 or 9 days. Data are mean ± SD from three independent experiments. (**c**) Relative expression of BCL11A and ZBTB7A in HUDEP-2 cells treated with indicated concentrations of hydroxyurea for 4 days. Data are mean ± SD from three independent experiments. (**d**) Relative expression of β- or γ-globin in hydroxyurea-treated BCL11A- or ZBTB7A-knockout cells. Data are mean ± SD from three independent experiments. (**e**) ChIP-qPCR analyses using antibodies against H3K9me2 in HUDEP-2 cells treated with 200 μM hydroxyurea or DMSO for 4 days. Data are mean ± SD from three independent experiments. *P*-value was calculated by one-way ANOVA with Tukey's post hoc test.

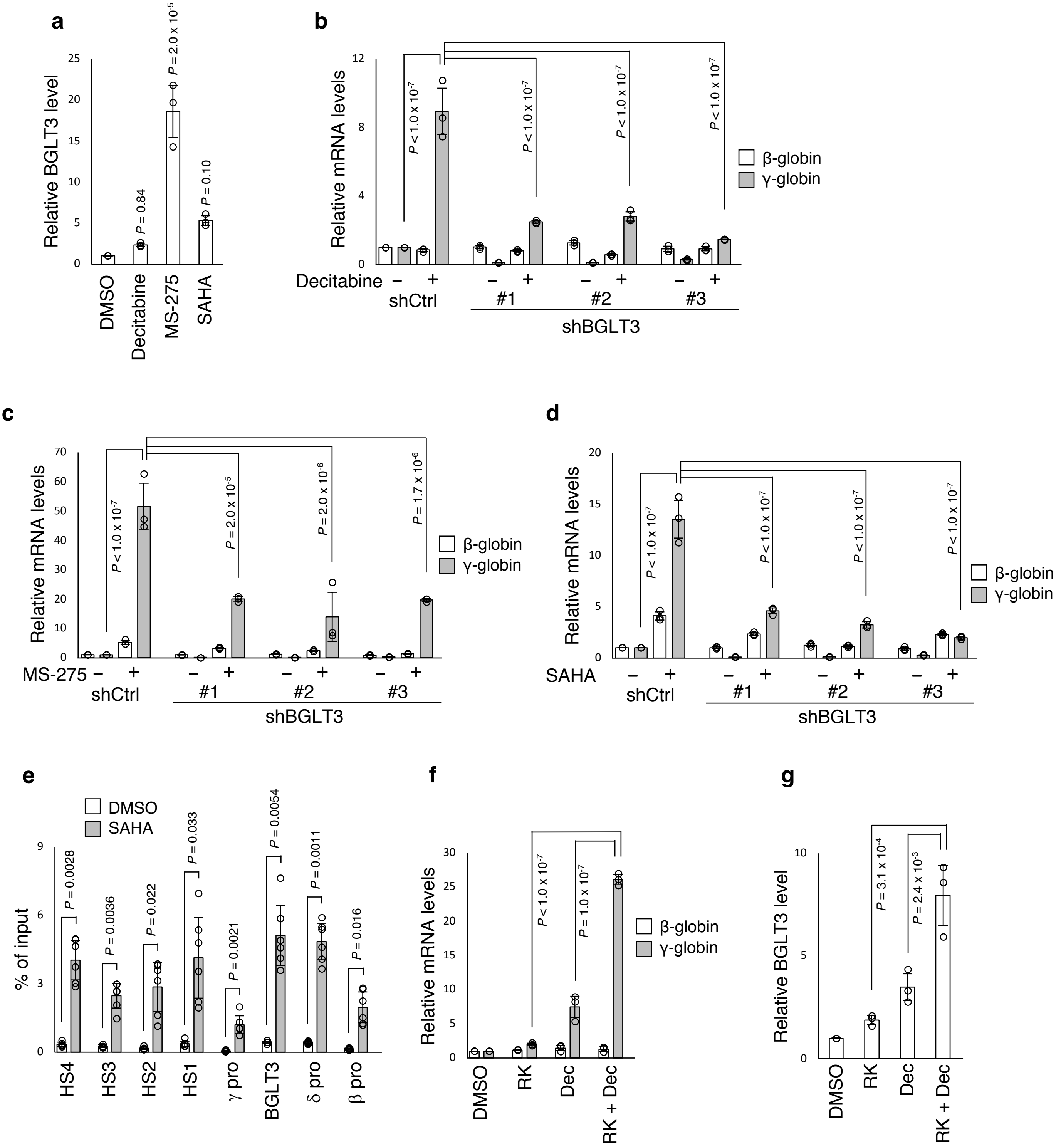

a
Relative BGLT3 level
0
5
10
15
20
25
P = 0.84
P = 2.0 x 10-5
P = 0.10
DMSO
Decitabine
MS-275
SAHA
b
Relative mRNA levels
0
4
8
12
P < 1.0 x 10-7
P < 1.0 x 10-7
P < 1.0 x 10-7
P < 1.0 x 10-7
β-globin
γ-globin
Decitabine – + – + – + – +
shCtrl
#1
#2
#3
shBGLT3
c
Relative mRNA levels
0
10
20
30
40
50
60
70
P < 1.0 x 10-7
P = 2.0 x 10-5
P = 2.0 x 10-6
P = 1.7 x 10-6
β-globin
γ-globin
MS-275 – + – + – + – +
shCtrl
#1
#2
#3
shBGLT3
d
Relative mRNA levels
0
5
10
15
20
P < 1.0 x 10-7
P < 1.0 x 10-7
P < 1.0 x 10-7
P < 1.0 x 10-7
β-globin
γ-globin
SAHA – + – + – + – +
shCtrl
#1
#2
#3
shBGLT3
e
DMSO
SAHA
% of input
0
3
6
9
P = 0.0028
P = 0.0036
P = 0.022
P = 0.033
P = 0.0021
P = 0.0054
P = 0.0011
P = 0.016
HS4
HS3
HS2
HS1
γ pro
BGLT3
δ pro
β pro
f
Relative mRNA levels
0
5
10
15
20
25
30
P < 1.0 x 10-7
P = 1.0 x 10-7
β-globin
γ-globin
DMSO
RK
Dec
RK + Dec
g
Relative BGLT3 level
0
5
10
P = 3.1 x 10-4
P = 2.4 x 10-3
DMSO
RK
Dec
RK + Dec

Supplementary Fig. 11. Involvement of BGLT3 in γ-globin induction by HbF inducers. (**a**) Relative expression of *BGLT3* in HUDEP-2 cells treated with the indicated 1 μM inhibitors for 3 days. Data are mean ± SD from three independent experiments. (**b**–**d**) Relative expression of β- and γ-globin genes in BGLT3-knockdown HUDEP-2 cells treated with 1 μM decitabine (b), 1 μM MS-275 (c), or 1 μM SAHA (d) for 3 days. Data are mean ± SD from three independent experiments. (**e**) ChIP-qPCR analyses using antibodies against H3K9ac in HUDEP-2 cells treated with 1 μM SAHA or DMSO for 1 day. Data are mean ± SD from three independent experiments. (**f** and **g**) Relative expression of β- and γ-globin (f) and BGLT3 (g) genes in HUDEP-2 cells treated with 1 μM RK-701 and decitabine for 3 days. Data are mean ± SD from three independent experiments. *P*-value was calculated by one-way ANOVA with Tukey's post hoc test except for (**e**), calculated by two-tailed *t*-test.

HBG1_exon3
BGLT3 sequence

1. 3670 nt (**bold**, Guo, G. et al. *Oncogene.* **34,** 1768-1779 (2015)., Accession#: KF110790)
2. 1684 nt (**magenta**, Ivaldi, M. S. et al. *Blood.* **132**, 1963-1973 (2018).)

[binding motif: **BCL11A**, **ZBTB7A**]

ctcctgggaaatgtgctggtgaccgttttggcaatccatttcggcaaagaattcacccctgaggtgcaggcttcctggcagaagatggtgactgcagtggc
cagtgccctgtcctccagataccactgagctcactgcccatgattcagagctttcaaggataggctttattctgcaagcaatacaaataataaatctattctg
ctgagagatcacacatgattttcttcagctcttttttttacatctttttaaatatatgag**ccacaaagggtttatattgagggaagtgtgtatgtgtatttctgc**
atgcctgtttgtgtttgtggtgtgtgcatgctcctcatttatttttatatgagatgtgcattttgatgagcaaataaaagcagtaaagacacttgtaca
cgggagttctgcaagtgggagtaaatggtgtaggagaaatccggtgggaagaaagacctctataggacaggacttctcagaaacagatgt
tttggaagagatgggaaaaggttcagtgaagacctgggggctggattgattgcagctgagtagcaaggatggttcttaaggaagggaaag
tgttccaagctttaggaattcaaggtttagtcaggtgtagcaattctattttattaggaggaatactatttctaatggcacttagcttttcacagccc
ttgtggatgcctaagaaagtgaaattaatcccatgccctcaagtgtgcagattggtcacagcatttcaagggagagacctcattgtaagac
tctgggggaggtggggacttaggtgtaagaaatgaatcagcagaggctcacaagtcagcatgagcatgttatgtctgagaaacagaccag
cactgtgagatcaaaatgtagtgggaagaatttgtacaacattaattggaaggcttacttaatggaatttttgtatagttggatgttagtgcatctc
tataagtaagagtttaatatgatggtgttacggacctaatgtttgtgtctcctcaaaattcacatgctgaatccccaactcccaactgaccttatct
gtgggggaggcttttgaaaagtaattaggtttagatgagctcataagagcagatccccatcataaaattattttccttatcagaagcagagaga
caagccatttctctttcctcccggtgaggacacagtgagaagtccgccatctgcaatccaggaagagaaccctgaccacgagtcagcct
tcagaaatgtgagaaaaaactctgttgttgaagccacccagtcttttgtattttgttatagcaccttgcactgagtaaggcagatgaagaagga
gaaaaaaataagcttgggttttgagtggactacagaccatgtttatctcaggtttgcaaagctcccctcgtcccctatgtttcagtataaaatac
ctactctactactctcatctataagacccaaataataagcctgcgcccttctctctaactttgatttctcctatttttacttcaacatgctttactcta
gccttgtaatgtctttacatacagtgaaatgtaaagttctttattctttttttcttctttcttttttctcctcagcctcagaatttggcacatgcccttcctt
ctttcaggaacttctccaacatctctgcctggctccatcatatcataaaggtcccacttcaaatgcagtcactaccgtttcagaatatgcactttc
tttctttttttgtttttttgttttttttaagtcaaagcaaatttcttgagagagtaaagaaataaacgaatgactactgcataggcagagcagccccgag
ggccgctggttgttccttttatggttatttcttgatgatatgttaaacaagttttggattatttatgccttctctttttaggccatatagggtaactttctg
acattgccatggcatttttcttttaatttaatttactgttaccttaaattcaggggtacacgtacaggatatgcaggtttgttttataggtaaaagtgtg
ccatggttttaatgggttttttttttcttgtaaagttgtttaagtttcttgtttactctggatattaggcctttgtcagaagaatagattggaaaatctttttc
ccattctgtagattgtctttcgctctgatggtagtttcttttgctgagcaggagctctttagtttaattagattccattggtcaattttgcttttgctgc
aattgcttttcacgctttcatcatgaaatctgtgcccgtgtttatatcatgaatagtattgccttgatttttttctaggctttttatagtttggggtttttcat
ttaagtctctaatccatctggagttaattttggataaggtataaggaaggagtccagtttcatttttcagcatatggctagccagttctccccccatc
atttattaaattgaaaatcctttccccattgcttgcttttgtcaggtttctaaaagaccagatggttgtaggtacaatatgcagtttcttcaagtcatat
aataccatctgaaatctcttattaattcatttcttttagtatgtatgctggtctcctctgctcactatagtgagggcaccattagccagagaatctgt
ctgtctagttcatgtaagattctcagaattaagaaaaatggatggcatatgaatgaaacttcatggatgacatatggaatctaatatgtatttgttg
aattaatgcataagatgcaacagagagaagttgacaactgcaatgataacctggtattgatgatataagagtctatagatcacagtagaagc
aataatcatggaaaacaattggaaatggggaacagccacaaacaagaaagaatcaatacttccaggaaagtgactgcaggtcacttttcct
ggagcgggtgagagaaaagtggaagttagcagtaactgctgaattcctggttggctgatggaaagatggggcagctgttcactggtacgc
agggttttagatgtatgtacctaaggatatgaggtatggcaatgaacagaaattcttttgggaatgagttttagggccattaaaggacatgacc
tgaagtttcctctgaggccagtccccacaactcaatataaatgtgtttcctgcatatagtcaaagttgccacttctttttcttcatatcatcgatctct
gctcttaaagataatcttggttttgcctcaaactgtttgtcactacaaactttccccatgttcctaagtaaaacaggtaactgcctctcaactatatc
aagtagactaaaatattgtgtctctaatatcagaaattcagctttaatatattgggtttaactctttgaaatttagagtctccttgaaatacacatgg
gggtgatttcctaaactttatttcttgtaaggatttatctcaggggtaacacacaaaccagcatcctgaacctctaagtatgaggacagtaagc
cttaagaatataaaataaactgttcttctctctgccggtggaagtgtgccctgtctattcctgaaattgcttgtttgagacgcatgagacgtgcag
cacatgagacacgtgcagcagcctgtggaatattgtcagtgaagaatgtctttgcctgattagatataaagacaagttaaacacagcattaga
ctatagatcaagcctgtgccagacacaaatgacctaatgcccagcacgggccacggaatctcctatcctcttgcttgaacagagcagcac
acttctcccccaacactattagatgttctggcataattttgtagatatgtaggatttgacatggactattgttcaatgattcagaggaaatctccttt
gttcagataagtacactgactactaaatggattaaaaaacacagtaataaaacccagttttcccctta

Supplementary Fig. 12 Recognition motifs of BCL11A and ZBTB7A in the BGLT3 gene locus.

The nucleic acid sequence of BGLT3 downstream of HBG1 is shown. Two predicted BGLT3 transcripts have been reported: 3670 nt (Accession# KF110790, black bold)[2] and 1684 nt (magenta background)[3]. Sequences predicted to be recognized by BCL11A[4] or ZBTB7A[5] are highlighted by a gray or red background, respectively. The HBG1 gene region (exon3) is shown in white on a black background.

Supplementary Fig. 13-1

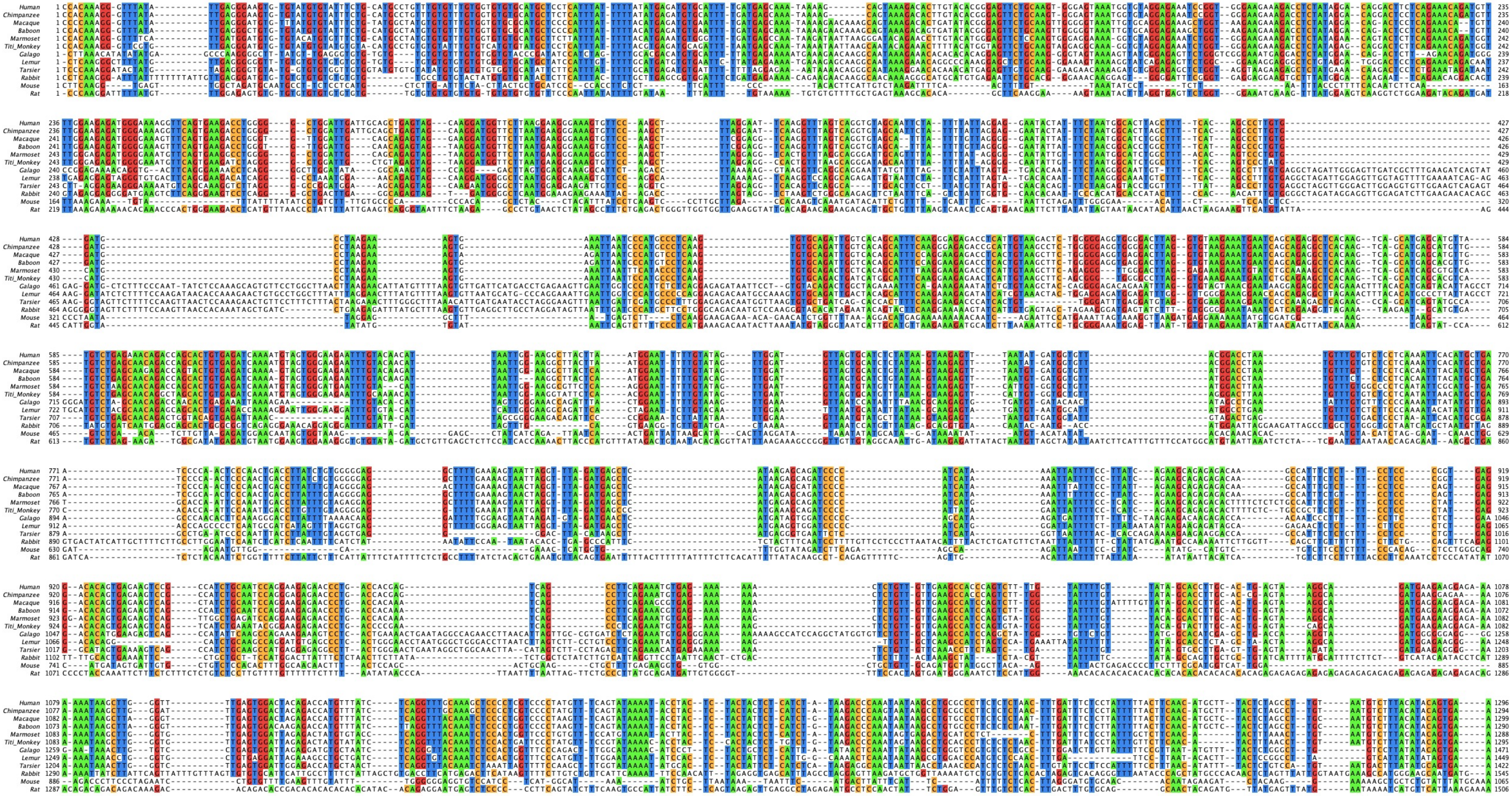

Supplementary Fig. 13-2

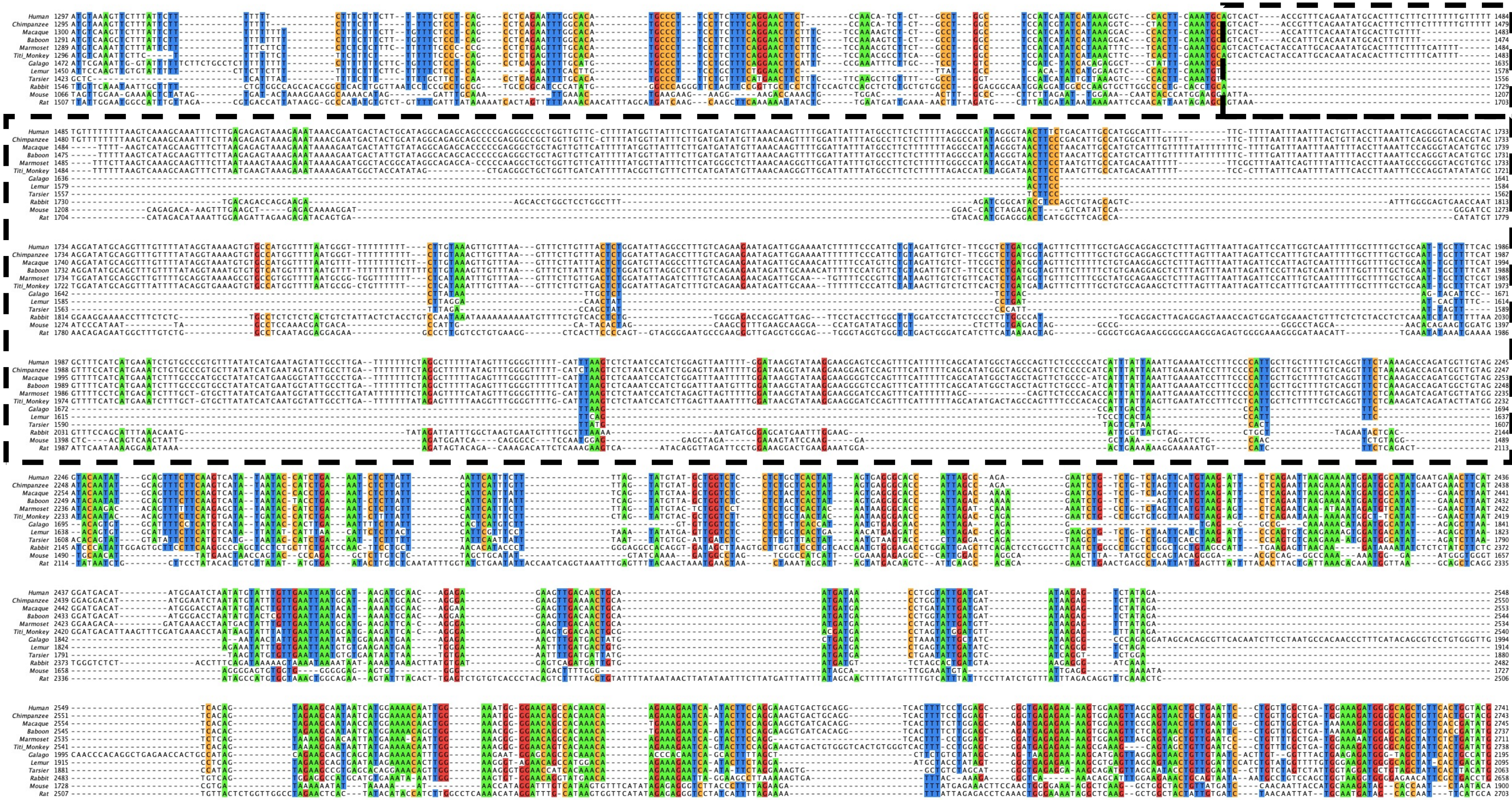

Supplementary Fig. 13-3

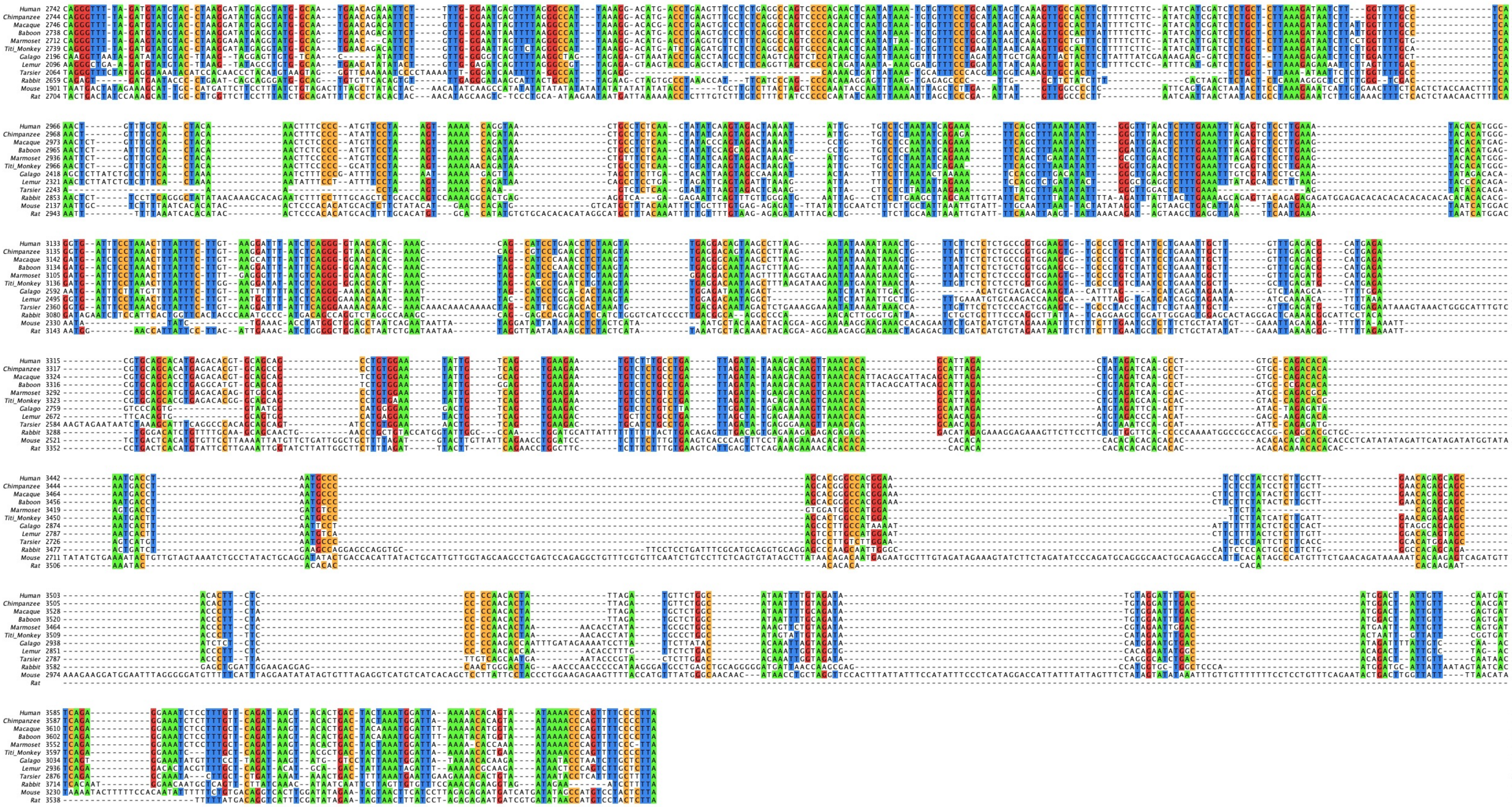

Supplementary Fig. 13 Multiple sequence alignment of the gene locus corresponding to BGLT3 in 12 mammalian species, including humans.

The predicted BGLT3 sequences of 11 mammalian species were obtained from the National Center for Biotechnology Information (NCBI) database, and were inferred based on the human BGLT3 sequence (3670 nt, see Supplementary Fig. 12). The alignment of 12 mammalian species was assessed by JalView. Bases with over 70% conservation in the alignment columns are colored (green: A, blue: T, red: G, and orange: C). The region enclosed by the dotted black line represents well-conserved sequences among Human, Chimpanzee, Macaque, Baboon, Marmoset, and Titi Monkey.

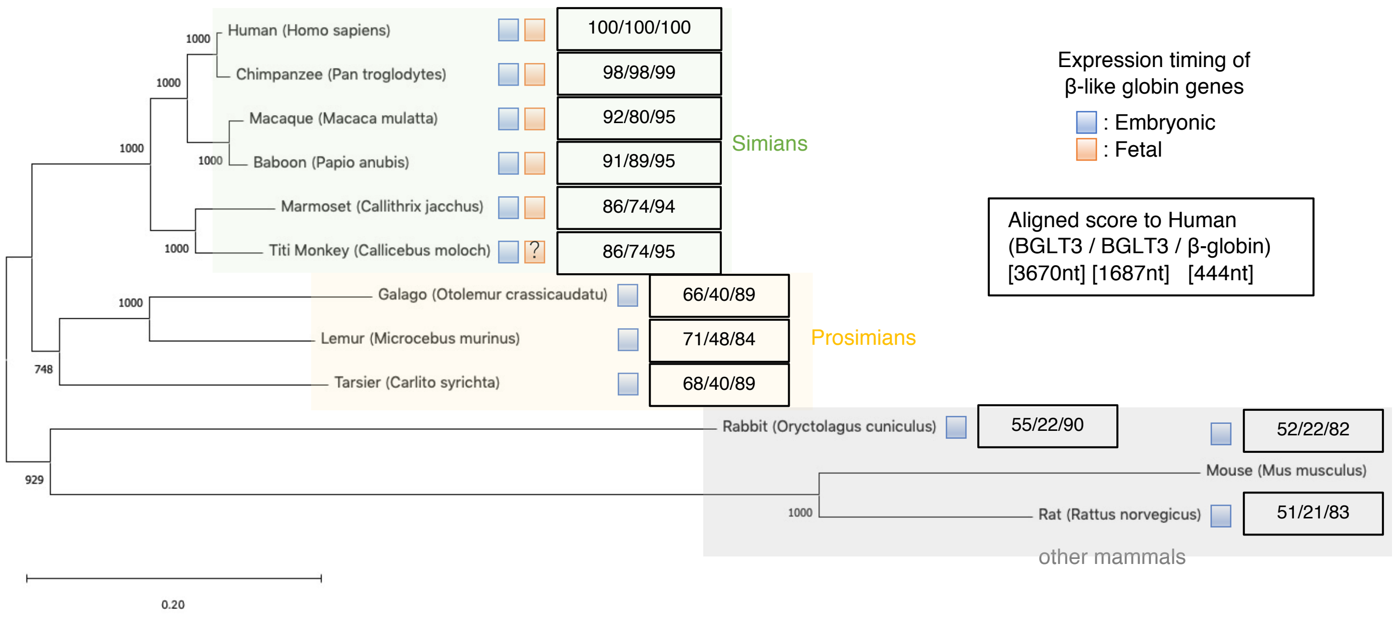

Supplementary Fig. 14 Conservation of BGLT3 sequences and expression timing of β-like globin among various mammals.

Molecular dendrogram of the BGLT3 gene and expression timing of the β-like globin gene. The phylogenetic tree was drawn based on the neighbor-joining (NJ) method (1,000 bootstrap repeats). The numbers above branches indicate bootstrap support values of the NJ method. The phylogenetic analysis was visualized using Molecular Evolutionary Genetics Analysis software. The expression timing of the β-like globin gene and the hierarchies of biologically relevant groupings are based on multiple studies[6-8]. Alignment scores between the human BGLT3 (long or short, see Supplementary Fig. 12 and 13) or β-globin gene and those in other organisms were evaluated using the default options of ClustalW.

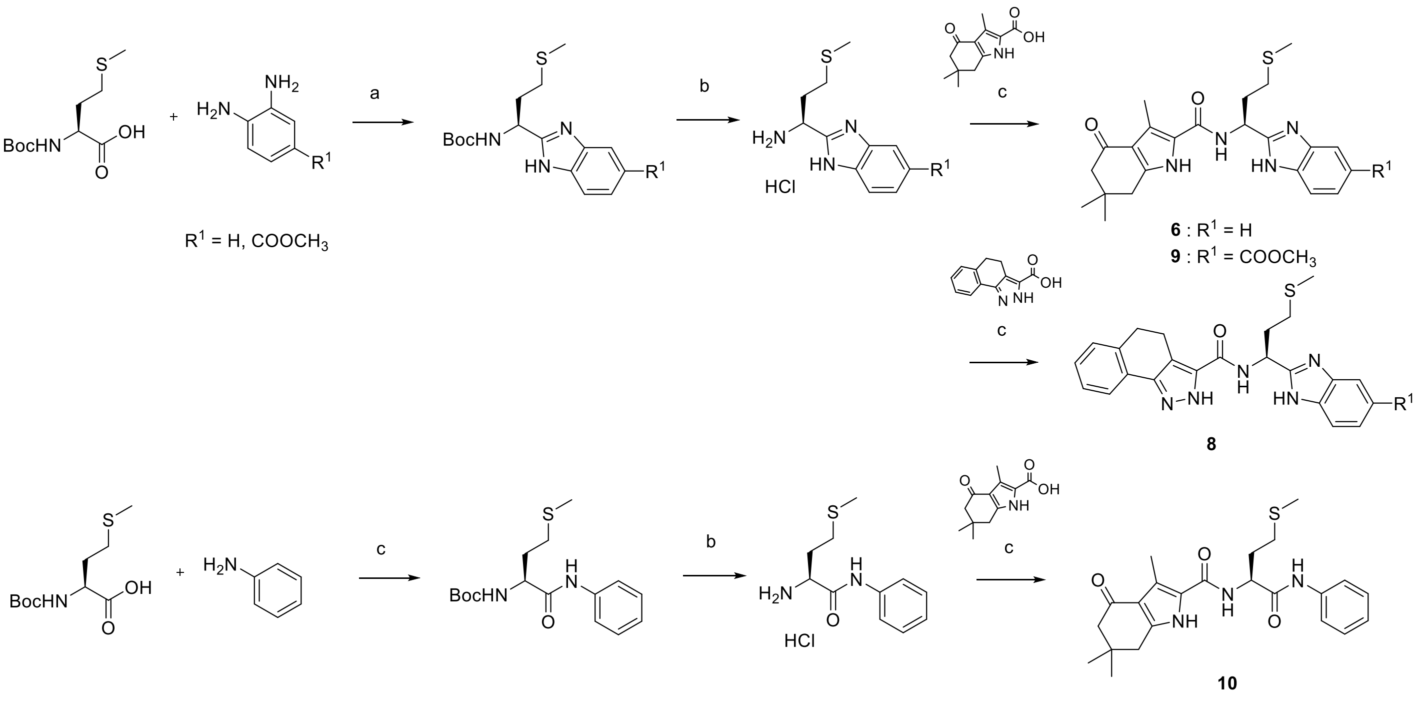

Supplementary Fig. 15. Reagents and conditions:

(a) EDCI-HCl, HOBt-H_2O, DIEA, DMF, rt; and then AcOH, 60°C ; (b) HCl/EtOAc, rt; (c) EDCI-HCl, HOBt-H_2O, DIEA, DMF, rt.

Abbreviations: Boc, *tert*-butoxycarbonyl; DIEA, *N*,*N*-diisopropylethylamine; DMF, *N*,*N*-dimethylformamide; EDCI, 1-ethyl-3-(3-dimethylaminopropyl) carbodiimide; HOBt, 1,2,3-benzotriazol-1-ol.

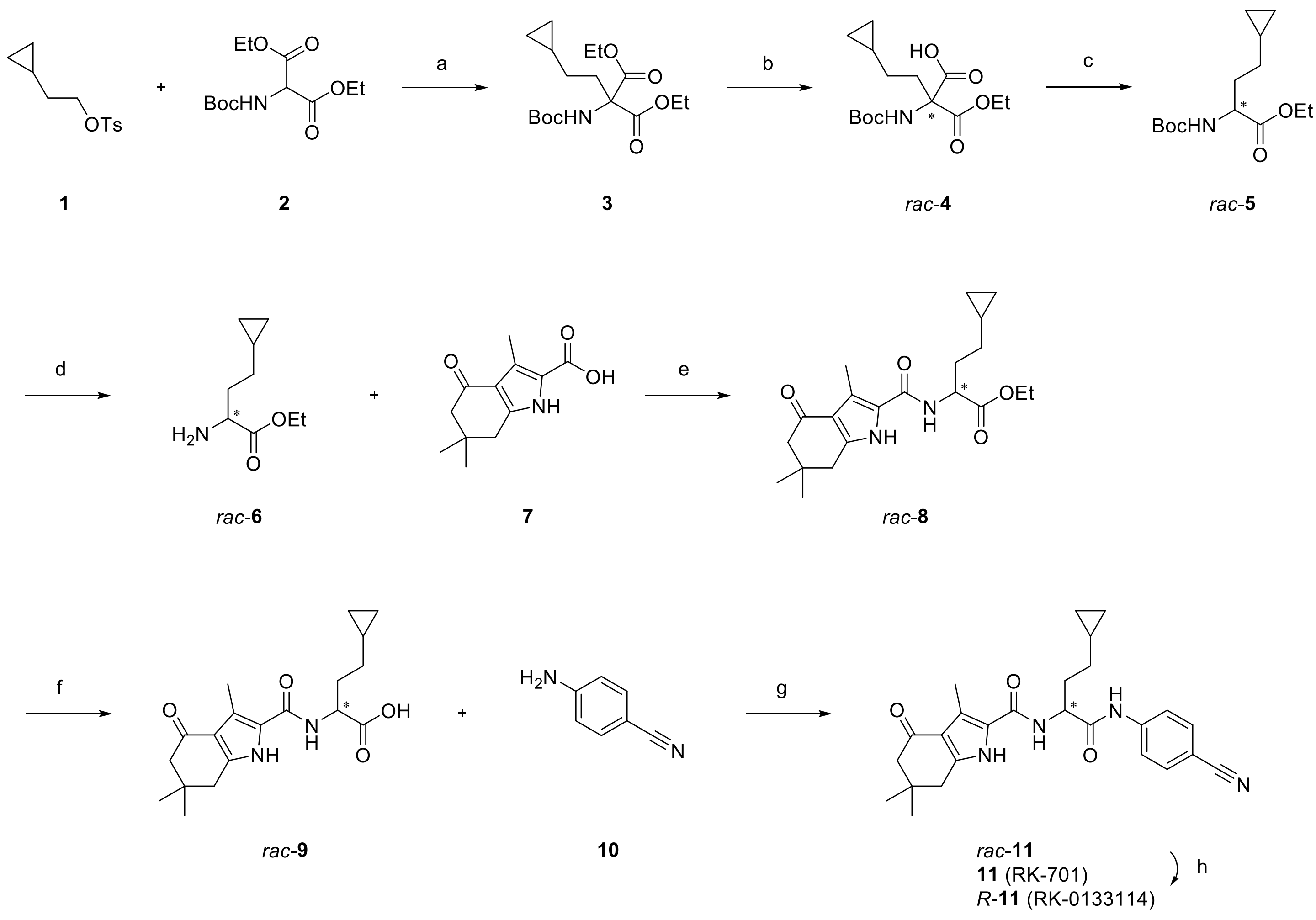

Supplementary Fig. 16 Reagents and conditions:

(a) *tert*-BuOK, DMF, 70° C; (b) aq. KOH, EtOH, rt; (c) 1,2-diClPh, 110° C; (d) TFA, CH_2Cl_2, rt; (e) EDCI-HCl, HOBt-H_2O, DIEA, DMF, rt; (f) LiOH-H_2O, EtOH/THF/H_2O, rt; (g) DMT-MM nH_2O, DMF, rt; (h) CHIRALPAK IA.

Abbreviations: Boc, *tert*-butoxycarbonyl; DIEA, *N*,*N*-diisopropylethylamine; DMF, *N*,*N*-dimethylformamide; DMT-MM, 4-(4,6-dimethoxy-1,3,5-triazin-2-yl)-4-methylmorpholinium chloride; EDCI, 1-ethyl-3-(3-dimethylaminopropyl) carbodiimide; HOBt, 1,2,3-benzotriazol-1-ol; TFA, trifluoroacetic acid; THF, tetrahydrofuran; Ts, *p*-toluenesulfonyl.

Supplementary Table 1. Structure-activity relationship of G9a inhibitors.
G9a activity was measured by the AlphaLISA-based assay. Data are mean ± SD from three independent experiments.

Compound	Structure	IC50 (μM)
1	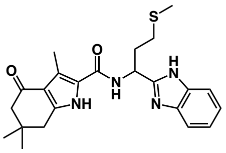	5.3 ± 1.0 μM
2	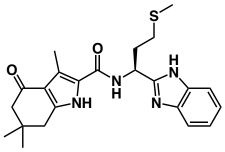	1.4 ± 0.082 μM
3	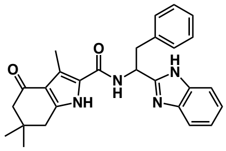	> 100 μM
4	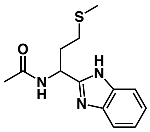	> 100 μM
5	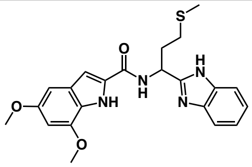	> 100 μM
6	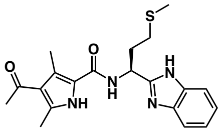	88.2 ± 0.89 μM
7	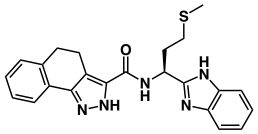	> 50 μM
8	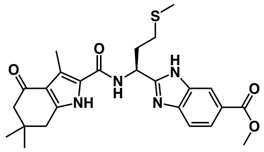	21.3 ± 3.0 μM
9	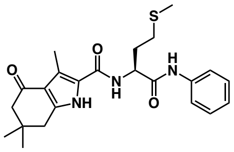	0.30 ± 0.013 μM
10 (RK-701)	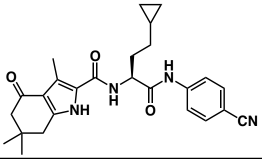	0.027 ± 0.0023 μM

Supplementary Table 2. Selectivity against 4 DNA methyltransferases and 33 methyltransferases.

Compound was tested in a 5-dose IC50 mode with 10-fold serial dilution, in duplicate, at 10 or 100 µM. Control compounds, SAH (S-(5'-Adenosyl)-L-homocysteine) and Chaetocin, were tested in 10-dose IC50 mode with 3-fold serial dilution starting at 100 or 200 µM. Reactions were carried out at 1 µM SAM. Curve fits were performed where the enzyme activities at the highest concentration of compounds were less than 65%. ND indicates compound not tested against enzyme.

Methyltransferase	IC50 (µM)		
	RK-701	SAH	Chaetocin
ASH1L	79.4	ND	0.093
DNMT1	> 100	0.13	ND
DNMT3a	> 100	0.30	ND
DNMT3b	> 100	0.11	ND
DNMT3b/3L	> 100	0.05	ND
DOT1L	> 100	1.70	ND
EZH1	> 100	22.8	ND
EZH2	> 100	13.8	ND
EZH2 (Y641F)	> 100	64.1	ND
G9a	0.023	6.62	ND
GLP	0.053	3.86	ND
MLL1	> 100	1.3	ND
MLL2	> 100	11.6	ND
MLL3	> 100	49.7	ND
MLL4	> 100	1.1	ND
NSD1	> 100	56.6	ND
NSD2	> 100	3.3	ND
NSD2 (E1099K)	> 100	6.5	ND
NSD2 (T1150A)	> 100	7.2	ND
NSD3	> 100	ND	0.15
PRDM9	> 100	ND	13.4
PRMT1	> 100	0.26	ND
PRMT3	> 100	2.4	ND
PRMT4	> 100	0.18	ND
PRMT5/MEP50	> 100	1.2	ND
PRMT6	> 100	0.41	ND
PRMT7	> 100	0.11	ND
PRMT8	> 100	0.24	ND
SET1b	48.6	5.3	ND
SET7/9	> 100	81.8	ND
SET8	> 100	311.4	ND
SETD2	> 100	5.3	ND
SMYD1	52.7	47.4	ND
SMYD2	> 100	1.9	ND
SUV39H1	> 100	90.6	ND
SUV39H2	> 100	20.2	ND
SUV420H1TV2	> 100	125.7	ND

Supplementary Table 3. Data collection and refinement statistics for complex.

PDB ID	7X73
**Data Collection**	
Beamline	SLS X06DA
Wavelength (Å)	1.000
Space group	*P*1
Unit cell dimensions	
a, b, c (Å)	43.2, 56.2, 66.5
α, β, γ (°)	76.1, 72.5, 67.5
Resolution (Å)	42.90−1.49 (1.52−1.49) *
No. of unique reflections	86,112
Completeness (%)	96.6 (94.1)
Multiplicity	3.6 (3.5)
Mean $I/\sigma(I)$	15.1 (1.6)
R_{sym} (%)	4.8 (83.6)
R_{pim} (%)	3.0 (51.6)
$CC_{1/2}$	0.999 (0.705)
**Refinement**	
R_{work} (%)	16.7
R_{free} (%)	19.3
RMSD bond lengths (Å)	0.006
RMSD bond angles (°)	0.872
No. of reflections (total)	86,078
No. of reflections (test set)	4,183
No. of atoms	
Total	5,142
Protein	4,416
Inhibitor	66
Coenzyme	54
Water	578
Other	28
Mean *B*-factor (Å^2)	
Overall	30.7
Protein	30.3
Inhibitor	28.5
Coenzyme	22.0
Water	35.1
Other	32.9
Ramachandran plot (%)	
Favored	95.9
Allowed	4.1

*Values in parentheses are for the highest-resolution bin.

Supplementary Table 4. Biochemical property of RK-701.
A to B: Apical to Basal; B to A: Basal to Apical. Data of solubility, PAMPA, and Caco-2 permeability represent means ± SD from at least three independent experiments, while data of metabolic stability represent averages from two independent experiments.

Solubility in PBS (μM)	
pH6.8	26.3 ± 3.2
pH7.4	10.9 ± 2.2
PAMPA permeability ($x10^{-6}$ cm/s)	3.08 ± 1.47
Caco-2 permeability ($x10^{-6}$ cm/s)	
Papp A to B	5.23 ± 0.47
Papp B to A	18.9 ± 0.92
Efflux ratio (B to A/A to B)	3.6
Metabolic stability in liver microsomes CLint (mL/min/mg protein)	
Human	0.019
Monkey	0.067
Dug	0.028
Rat	0.033

Supplementary Table 5. Hematological and hepatic parameters of rats treated with RK-701 for two weeks.

RK-701 (mg/kg)	0	10	30	100
RBC (x 10^4 cells/μL)	721 ± 15	741 ± 19	726 ± 36	728 ±1 7
HGB (g/dL)	14.8 ± 0.5	15.1 ± 0.7	14.5 ± 0.5	14.8 ± 0.3
HCT (%)	42.6 ± 1.3	43.7± 1.7	42.6 ± 1.4	42.9 ± 1.1
MCV (fL)	59.2 ± 1.7	58.9± 1.6	58.7 ± 2.6	59.0 ± 0.7
MCH (pg)	20.5 ± 0.8	20.4 ± 0.6	20.0 ± 1.0	20.3 ± 0.2
MCHC (g/dL)	34.6 ± 0.8	34.6 ± 0.3	34.0 ± 0.5	34.4 ± 0.3
Retic (x 10^9 cells/L)	221 ± 30.5	190 ± 28.3	228 ± 37.5.	179 ± 45.9
PLT (x 10^4 cells/μL)	129 ± 16.5	128 ± 11.2.	122 ± 15.7	113 ± 10.8
WBC (x 10^2 cells/μL)	63.1 ± 19.4	59.8 ± 12.2	61.6 ± 9.2	74.8 ± 16.8
Lymph (x 10^2 cells/μL)	50.1 ± 14.5	51.4 ± 10.7	53.0 ± 10.3.	64.6 ± 14.9
NEUT (x 10^2 cells/μL)	10.6 ± 4.5	6.4 ± 1.7	6.7 ± 2.1	7.8 ± 2.9
EOS (x 10^2 cells/μL)	0.8 ± 0.2	0.6 ± 0.1	0.5 ± 0.1	0.6 ± 0.2
MONO (x 10^2 cells/μL)	1.2 ± 0.9	0.9 ± 0.2	0.9 ± 0.1	1.2 ± 0.4
LUC (x 10^2 cells/μL)	0.4 ± 0.2	0.4 ± 0.2	0.4 ± 0.1	0.5 ± 0.2
AST (IU/L)	58 ± 5	64 ± 4	64 ± 7	64 ± 5
ALT (IU/L)	25 ± 1	28 ± 5	26 ± 3	29 ± 5
ALP (IU/L)	504 ± 152	487 ± 170	511 ± 141	566 ± 181
T-CHO (mg/dL)	85 ± 12	68 ± 13	64 ± 5	88 ± 20
TG (mg/dL)	28 ± 17	24 ± 15	17 ± 6	19 ± 10
PL (mg/dL)	143 ± 19	121 ± 18	117 ± 11	137 ± 26
TP (g/dL)	6.1 ± 0.2	6.0 ± 0.3	5.9 ± 0.2	5.8 ± 0.2
ALB (g/dL)	3.6 ± 0.1	3.6 ± 0.2	3.5 ± 0.2	3.4 ± 0.1
A/G	1.4 ± 0.1	1.5 ± 0.1	1.4 ± 0.0	1.4 ± 0.1

RBC: red blood cell, HGB: hemoglobin, HCT: hematocrit, MCV: mean corpuscular volume, MCH: mean corpuscular hemoglobin, MCHC: mean corpuscular hemoglobin concentration, Retic: reticulocyte, PLT: platelet, WBC: white blood cell, Lymph: lymphocyte, NEUT: neutrophil, EOS: eosinophil, MONO: monocyte, LUC: large unstained cell, AST: aspartate aminotransferase, ALT: alanine aminotransferase, T-CHO: total cholesterol, TG: triglyceride, PL: phospholipid, TP: total protein, ALB: albumin, A/G: albumin/globulin

Supplementary Table 6-1. List of the upregulated or downregulated genes by treatment with RK-701.

Regulation: Up			
Symbol	Description	log2FoldChange	LOG10(qvalue)
TSPO2	translocator protein 2(TSPO2)	1.00	4.13
ZNF440	zinc finger protein 440(ZNF440)	1.02	2.24
CCDC71L	coiled-coil domain containing 71 like(CCDC71L)	1.02	13.51
ULBP1	UL16 binding protein 1(ULBP1)	1.02	18.78
RNASE1	ribonuclease A family member 1, pancreatic(RNASE1)	1.02	1.61
QSOX1	quiescin sulfhydryl oxidase 1(QSOX1)	1.03	11.93
RAET1K	retinoic acid early transcript 1K pseudogene(RAET1K)	1.03	1.47
SAMD9	sterile alpha motif domain containing 9(SAMD9)	1.04	17.55
SMIM14	small integral membrane protein 14(SMIM14)	1.06	2.66
C10orf25	ZNF22 antisense RNA 1(ZNF22-AS1)	1.06	3.17
RNLS	renalase, FAD dependent amine oxidase(RNLS)	1.06	1.90
BSN	bassoon presynaptic cytomatrix protein(BSN)	1.07	1.74
FCGR2A	Fc gamma receptor IIa(FCGR2A)	1.07	2.92
CR1L	complement C3b/C4b receptor 1 like(CR1L)	1.07	3.46
CD53	CD53 molecule(CD53)	1.08	56.35
RP11-10L12.4		1.08	1.32
AC006116.17		1.11	7.90
PASD1	PAS domain containing repressor 1(PASD1)	1.11	3.86
FCGR2C		1.15	3.17
GPR65	G protein-coupled receptor 65(GPR65)	1.15	1.55
RP11-420K14.1		1.18	25.55
FRY	FRY microtubule binding protein(FRY)	1.18	4.91
CD84	CD84 molecule(CD84)	1.19	25.57
CPS1	carbamoyl-phosphate synthase 1(CPS1)	1.20	12.28
CAPS2	calcyphosine 2(CAPS2)	1.21	4.37
FMO4	flavin containing dimethylaniline monoxygenase 4(FMO4)	1.23	1.37
HBG1	hemoglobin subunit gamma 1(HBG1)	1.23	9.03
TTN	titin(TTN)	1.24	2.92
PRG2	proteoglycan 2, pro eosinophil major basic protein(PRG2)	1.24	23.43
SEPP1	selenoprotein P(SELENOP)	1.26	19.42
SYP	synaptophysin(SYP)	1.26	4.83
AKR1C1	aldo-keto reductase family 1 member C1(AKR1C1)	1.26	5.56
AC006116.22		1.27	2.46
TMEM74	transmembrane protein 74(TMEM74)	1.29	1.36
ATP1B1	ATPase Na+/K+ transporting subunit beta 1(ATP1B1)	1.29	1.71
DZIP3	DAZ interacting zinc finger protein 3(DZIP3)	1.30	29.50
SLC8A3	solute carrier family 8 member A3(SLC8A3)	1.33	2.72
GS1-122H1.2		1.33	6.02
EFCAB10	EF-hand calcium binding domain 10(EFCAB10)	1.33	5.94
CPA3	carboxypeptidase A3(CPA3)	1.33	2.94
TMEM144	transmembrane protein 144(TMEM144)	1.34	5.19
TMEM133		1.37	4.87
HSPA7	heat shock protein family A (Hsp70) member 7 (pseudogene)(HSPA7)	1.37	7.04
RHD	Rh blood group D antigen(RHD)	1.38	5.17
TRIM10	tripartite motif containing 10(TRIM10)	1.38	23.67
XAF1	XIAP associated factor 1(XAF1)	1.39	2.71
RP11-38M8.1	uncharacterized LOC105375519(LOC105375519)	1.39	1.50
ZNF365	zinc finger protein 365(ZNF365)	1.41	3.41
RP11-538D16.2		1.42	2.81
KRT79	keratin 79(KRT79)	1.42	2.79
PTPRC	protein tyrosine phosphatase receptor type C(PTPRC)	1.44	22.86
FPR3	formyl peptide receptor 3(FPR3)	1.45	5.06
MYO5B	myosin VB(MYO5B)	1.45	9.15
RP11-284F21.10		1.49	1.72
UTRN	utrophin(UTRN)	1.50	15.52
NID1	nidogen 1(NID1)	1.52	2.31
ARHGAP25	Rho GTPase activating protein 25(ARHGAP25)	1.52	2.49
ATP8A2	ATPase phospholipid transporting 8A2(ATP8A2)	1.55	1.40
IGSF3	immunoglobulin superfamily member 3(IGSF3)	1.56	3.06
CDC42EP3	CDC42 effector protein 3(CDC42EP3)	1.58	8.64
HLA-DMA	major histocompatibility complex, class II, DM alpha(HLA-DMA)	1.58	3.56
ALDH1A1	aldehyde dehydrogenase 1 family member A1(ALDH1A1)	1.58	12.98
HEPACAM2	HEPACAM family member 2(HEPACAM2)	1.60	8.09
CCDC64	BICD family like cargo adaptor 1(BICDL1)	1.60	3.45
DPYD	dihydropyrimidine dehydrogenase(DPYD)	1.61	2.02
NXF3	nuclear RNA export factor 3(NXF3)	1.63	17.99

Supplementary Table 6-2. List of the upregulated or downregulated genes by treatment with RK-701.

DISP2	dispatched RND transporter family member 2(DISP2)	1.66	10.79
LINC01033	long intergenic non-protein coding RNA 1033(LINC01033)	1.66	4.97
FMN1	formin 1(FMN1)	1.67	9.10
RUNX2	RUNX family transcription factor 2(RUNX2)	1.68	1.68
AKR1C2	aldo-keto reductase family 1 member C2(AKR1C2)	1.68	8.64
CTD-2643I7.1	beta globin locus transcript 3(BGLT3)	1.72	3.84
HBG2	hemoglobin subunit gamma 2(HBG2)	1.73	56.78
ADAMTS19	ADAM metallopeptidase with thrombospondin type 1 motif 19(ADAMTS19)	1.73	3.39
MEGF6	multiple EGF like domains 6(MEGF6)	1.74	10.62
ARHGAP42	Rho GTPase activating protein 42(ARHGAP42)	1.75	43.03
PTH2R	parathyroid hormone 2 receptor(PTH2R)	1.75	48.29
PDE4D	phosphodiesterase 4D(PDE4D)	1.76	8.07
EGFL6	EGF like domain multiple 6(EGFL6)	1.76	7.05
CASP4	caspase 4(CASP4)	1.78	26.02
TMPRSS11F	transmembrane serine protease 11F(TMPRSS11F)	1.79	1.43
PARK2	parkin RBR E3 ubiquitin protein ligase(PRKN)	1.83	12.43
C1orf115	chromosome 1 open reading frame 115(C1orf115)	1.85	12.66
CRISP2	cysteine rich secretory protein 2(CRISP2)	1.86	12.07
LRRC16B	capping protein regulator and myosin 1 linker 3(CARMIL3)	1.87	2.84
FAM46D	terminal nucleotidyltransferase 5D(TENT5D)	1.89	1.58
SCAMP5	secretory carrier membrane protein 5(SCAMP5)	1.97	2.39
RAB27B	RAB27B, member RAS oncogene family(RAB27B)	2.01	81.55
RP11-495P10.8		2.01	11.73
RP11-79H23.3		2.01	1.65
SP140L	SP140 nuclear body protein like(SP140L)	2.02	7.52
LINC00570	long intergenic non-protein coding RNA 570(LINC00570)	2.02	6.02
GCSAM	germinal center associated signaling and motility(GCSAM)	2.02	2.66
MACC1	MET transcriptional regulator MACC1(MACC1)	2.03	23.88
PIFO	primary cilia formation(PIFO)	2.04	1.89
RUNDC3A	RUN domain containing 3A(RUNDC3A)	2.04	4.00
LTF	lactotransferrin(LTF)	2.05	2.31
AGMO	alkylglycerol monooxygenase(AGMO)	2.07	1.65
SCIN	scinderin(SCIN)	2.08	83.47
KIF4B	kinesin family member 4B(KIF4B)	2.10	17.21
CARD16	caspase recruitment domain family member 16(CARD16)	2.12	5.39
FCGR2B	Fc gamma receptor IIb(FCGR2B)	2.14	1.80
TRAF3IP3	TRAF3 interacting protein 3(TRAF3IP3)	2.15	3.77
RP11-180K7.1		2.16	4.03
HBE1	hemoglobin subunit epsilon 1(HBE1)	2.16	4.40
SIGLEC22P		2.27	2.62
SYN1	synapsin I(SYN1)	2.29	22.41
ABLIM3	actin binding LIM protein family member 3(ABLIM3)	2.35	2.99
TENM1	teneurin transmembrane protein 1(TENM1)	2.38	5.69
FCER1A	Fc epsilon receptor Ia(FCER1A)	2.40	3.18
PLXNA4	plexin A4(PLXNA4)	2.45	5.01
HEPH	hephaestin(HEPH)	2.45	2.89
RP11-354E11.2	uncharacterized LOC101928834(LOC101928834)	2.48	4.85
ABI3BP	ABI family member 3 binding protein(ABI3BP)	2.52	3.05
CSMD2	CUB and Sushi multiple domains 2(CSMD2)	2.55	9.82
RP11-495P10.7		2.63	26.31
SPRR2F	small proline rich protein 2F(SPRR2F)	2.63	11.68
GAPT	GRB2 binding adaptor protein, transmembrane(GAPT)	2.63	9.66
RP1-80N2.2		2.66	2.20
LINC01028	long intergenic non-protein coding RNA 1028(LINC01028)	2.68	1.42
IL18RAP	interleukin 18 receptor accessory protein(IL18RAP)	2.70	38.72
AC008069.1		2.72	4.12
RP11-553A10.1		2.79	4.42
RP11-618I10.1		2.82	2.17
COL4A4	collagen type IV alpha 4 chain(COL4A4)	2.85	2.28
NPM1P50	nucleophosmin 1 pseudogene 50(NPM1P50)	2.88	1.37
NR5A2	nuclear receptor subfamily 5 group A member 2(NR5A2)	2.89	18.86
KCNT2	potassium sodium-activated channel subfamily T member 2(KCNT2)	2.93	22.19
IFI16	interferon gamma inducible protein 16(IFI16)	3.05	28.76
RP11-63D14.1		3.06	9.66
HCLS1	hematopoietic cell-specific Lyn substrate 1(HCLS1)	3.08	32.20
TBXAS1	thromboxane A synthase 1(TBXAS1)	3.09	10.03
NCKAP5	NCK associated protein 5(NCKAP5)	3.12	26.20
RP3-460G2.2		3.14	2.00

Supplementary Table 6-3. List of the upregulated or downregulated genes by treatment with RK-701.

SH2D4B	SH2 domain containing 4B(SH2D4B)	3.15	1.34
FYB	FYN binding protein 1(FYB1)	3.16	4.73
AF127936.3		3.20	34.86
OR2AT4	olfactory receptor family 2 subfamily AT member 4(OR2AT4)	3.23	2.94
LAMB3	laminin subunit beta 3(LAMB3)	3.33	22.70
RP11-34P13.7		3.39	1.88
MTUS1	microtubule associated scaffold protein 1(MTUS1)	3.44	2.91
LINC00426	long intergenic non-protein coding RNA 426(LINC00426)	3.54	2.22
CASP1	caspase 1(CASP1)	3.55	4.48
RNASE6	ribonuclease A family member k6(RNASE6)	3.70	1.55
CHIAP3		3.70	1.55
RP11-208K4.1		3.72	1.66
MS4A2	membrane spanning 4-domains A2(MS4A2)	3.76	39.84
AC064834.1		3.77	6.96
ZNF827	zinc finger protein 827(ZNF827)	3.83	6.02
CTC-546K23.1		3.86	6.27
AC011891.5		3.88	2.00
AC092642.1		3.88	10.97
NECAB1	N-terminal EF-hand calcium binding protein 1(NECAB1)	4.01	2.39
RP11-861A13.4	long intergenic non-protein coding RNA 1215(LINC01215)	4.10	6.22
SMPD3	sphingomyelin phosphodiesterase 3(SMPD3)	4.11	5.05
AC003090.1	long intergenic non-protein coding RNA 3007(LINC03007)	4.12	2.65
AF127936.5		4.32	1.39
LINC01088	long intergenic non-protein coding RNA 1088(LINC01088)	4.39	5.84
RP11-1112C15.1	long intergenic non-protein coding RNA 1602(LINC01602)	4.49	1.60
GLRA3	glycine receptor alpha 3(GLRA3)	4.49	1.60
NLRP11	NLR family pyrin domain containing 11(NLRP11)	4.50	3.35
SPRR2E	small proline rich protein 2E(SPRR2E)	4.54	1.71
TDRG1	testis development related 1(TDRG1)	4.60	1.63
C12orf79	long intergenic non-protein coding RNA 1619(LINC01619)	4.62	2.80
PART1	prostate androgen-regulated transcript 1(PART1)	4.62	1.85
DDIT4L	DNA damage inducible transcript 4 like(DDIT4L)	4.74	2.25
CD300C	CD300c molecule(CD300C)	4.82	2.36
CD2	CD2 molecule(CD2)	5.01	2.89
CTD-2215E18.1		5.06	2.48
NEBL	nebulette(NEBL)	5.41	9.48
PRKG2	protein kinase cGMP-dependent 2(PRKG2)	5.55	5.01
GABRG1	gamma-aminobutyric acid type A receptor subunit gamma1(GABRG1)	5.62	4.71
RP11-11N9.4	uncharacterized LOC105379362(LOC105379362)	5.87	5.17
RP11-704M14.1	uncharacterized LOC105377267(LOC105377267)	6.22	7.47
RYR2	ryanodine receptor 2(RYR2)	7.26	183.22
MYO16	myosin XVI(MYO16)	Inf	10.81
SEMA3C	semaphorin 3C(SEMA3C)	Inf	1.63
LRP2	LDL receptor related protein 2(LRP2)	Inf	4.85
SGCG	sarcoglycan gamma(SGCG)	Inf	1.64
DKK1	dickkopf WNT signaling pathway inhibitor 1(DKK1)	Inf	1.61
TCN1	transcobalamin 1(TCN1)	Inf	5.59
ABCA8	ATP binding cassette subfamily A member 8(ABCA8)	Inf	1.50
GPA33	glycoprotein A33(GPA33)	Inf	1.37
TMEM140	transmembrane protein 140(TMEM140)	Inf	2.90
PTPN14	protein tyrosine phosphatase non-receptor type 14(PTPN14)	Inf	5.13
TMPRSS15	transmembrane serine protease 15(TMPRSS15)	Inf	1.64
INHBB	inhibin subunit beta B(INHBB)	Inf	1.90
CCL23		Inf	1.62
C15orf27	transmembrane protein 266(TMEM266)	Inf	1.64
UGT2B11	UDP glucuronosyltransferase family 2 member B11(UGT2B11)	Inf	3.77
XX-FW81066F1.2		Inf	1.63
RP5-937E21.8		Inf	8.42
AC016730.1		Inf	1.50
AP000431.2		Inf	2.05
AC027119.1		Inf	1.85
RP11-462G2.1		Inf	2.76
LINC00504	long intergenic non-protein coding RNA 504(LINC00504)	Inf	19.54
ADAMTS19-AS1	ADAMTS19 antisense RNA 1(ADAMTS19-AS1)	Inf	1.64
RP11-265F19.1		Inf	3.06
CTD-2353F22.1	uncharacterized LOC107986412(LOC107986412)	Inf	2.59
RP11-466P24.6		Inf	2.30
RP11-708B6.2		Inf	1.64
RP11-284F21.8		Inf	1.64
EMR4P		Inf	2.05

Supplementary Table 6-4 List of the upregulated or downregulated genes by treatment with RK-701.

Regulation: Down			
Symbol	Name	log2FoldChange	LOG10(qvalue)
TMEM176A	transmembrane protein 176A(TMEM176A)	-1.36	7.63
MLXIPL	MLX interacting protein like(MLXIPL)	-1.34	5.02
ZNF582	zinc finger protein 582(ZNF582)	-1.08	1.79
CELSR1	cadherin EGF LAG seven-pass G-type receptor 1(CELSR1)	-1.75	9.97
ZNF586	zinc finger protein 586(ZNF586)	-2.32	6.90
CERS4	ceramide synthase 4(CERS4)	-1.11	5.39
NINL	ninein like(NINL)	-1.21	1.42
RNF125	ring finger protein 125(RNF125)	-1.13	1.57
NDRG4	NDRG family member 4(NDRG4)	-1.15	2.64
SLC5A5	solute carrier family 5 member 5(SLC5A5)	-1.08	1.88
TMEM176B	transmembrane protein 176B(TMEM176B)	-1.12	10.15
AKNA	AT-hook transcription factor(AKNA)	-1.59	1.79
MYRF	myelin regulatory factor(MYRF)	-1.57	5.49
LPPR3	phospholipid phosphatase related 3(PLPPR3)	-2.88	5.95
TNNI3	troponin I3, cardiac type(TNNI3)	-1.48	2.67
HBZ	hemoglobin subunit zeta(HBZ)	-1.67	8.63
ZNF426	zinc finger protein 426(ZNF426)	-1.19	7.15
GFPT2	glutamine-fructose-6-phosphate transaminase 2(GFPT2)	-1.56	2.46
ZNF304	zinc finger protein 304(ZNF304)	-1.07	7.21
HSPA12B	heat shock protein family A (Hsp70) member 12B(HSPA12B)	-1.88	15.49
DYSF	dysferlin(DYSF)	-1.18	2.73
ZFP14	ZFP14 zinc finger protein(ZFP14)	-2.84	1.69
CILP2	cartilage intermediate layer protein 2(CILP2)	-1.32	4.99
ZNF761	zinc finger protein 761(ZNF761)	-1.03	4.09
COX6B2	cytochrome c oxidase subunit 6B2(COX6B2)	-1.08	2.17
FGFR4	fibroblast growth factor receptor 4(FGFR4)	-1.24	1.98
PTGER1	prostaglandin E receptor 1(PTGER1)	-2.42	1.34
AMN	amnion associated transmembrane protein(AMN)	-2.62	3.20
SLC38A8	solute carrier family 38 member 8(SLC38A8)	-1.89	8.16
ZNF229		-2.67	3.71
PLIN4	perilipin 4(PLIN4)	-1.67	3.37
KLK1	kallikrein 1(KLK1)	-1.11	2.59
TMC8	transmembrane channel like 8(TMC8)	-1.17	1.68
ZNF160	zinc finger protein 160(ZNF160)	-1.32	4.00
SPTBN2	spectrin beta, non-erythrocytic 2(SPTBN2)	-1.07	6.17
LCN15	lipocalin 15(LCN15)	-1.13	1.48
STAP2	signal transducing adaptor family member 2(STAP2)	-1.27	2.16
HKR1	zinc finger protein 875(ZNF875)	-1.40	1.42
ZNF320	zinc finger protein 320(ZNF320)	-1.00	2.28
ANO9	anoctamin 9(ANO9)	-2.05	4.78
PRSS57	serine protease 57(PRSS57)	-1.33	3.89
ZNF559	zinc finger protein 559(ZNF559)	-1.49	9.83
ZNF548	zinc finger protein 548(ZNF548)	-1.14	2.43
SH2D5	SH2 domain containing 5(SH2D5)	-1.73	2.01
ZNF569	zinc finger protein 569(ZNF569)	-1.84	3.12
ZNF136	zinc finger protein 136(ZNF136)	-1.86	1.52
ZNF441	zinc finger protein 441(ZNF441)	-1.08	3.47
ZNF420	zinc finger protein 420(ZNF420)	-1.09	3.99
ZNF772	zinc finger protein 772(ZNF772)	-1.74	5.49
CFD	complement factor D(CFD)	-1.10	2.92
ZNF813	zinc finger protein 813(ZNF813)	-1.66	2.90
ZNF583	zinc finger protein 583(ZNF583)	-1.06	3.67
ZNF808	zinc finger protein 808(ZNF808)	-1.02	2.43
ZNF525	zinc finger protein 525(ZNF525)	-1.02	6.19
SOX18	SRY-box transcription factor 18(SOX18)	-3.14	2.57
ZNF814	zinc finger protein 814(ZNF814)	-1.05	3.00
ZNF551	zinc finger protein 551(ZNF551)	-1.64	6.12
ZNF611	zinc finger protein 611(ZNF611)	-1.71	4.80
ZNF134	zinc finger protein 134(ZNF134)	-1.62	6.71
ZNF845	zinc finger protein 845(ZNF845)	-1.32	9.54
CTD-2006C1.2	ZNF433 and ZNF878 antisense RNA 1(ZNF433-AS1)	-1.10	2.05
CERS1	ceramide synthase 1(CERS1)	-1.59	4.44
RP3-395M20.8		-1.10	1.47
ZNF10	zinc finger protein 10(ZNF10)	-1.13	7.05
ZNF350	zinc finger protein 350(ZNF350)	-1.14	3.58

Supplementary Table 7. List of the expression level of genes related to globin switching.

	Symbol	Description	FoldChange	
			RK-701	shG9a
Globin	HBB	hemoglobin beta	1.13 ± 0.05	1.05 ± 0.18
	HBBP1	hemoglobin beta pseudogene 1	0.99 ± 0.02	2.48 ± 1.76
	HBD	hemoglobin delta	1.01 ± 0.03	1.10 ± 0.59
	HBE1	hemoglobin epsilon 1	4.70 ± 0.71	8.19 ± 3.52
	HBG1	hemoglobin gamma A	2.51 ± 0.58	97.81 ± 94.48
	HBG2	hemoglobin gamma G	3.33 ± 0.28	46.70 ± 28.27
	HBA1	hemoglobin alpha 1	1.15 ± 0.13	1.89 ± 0.46
	HBA2	hemoglobin alpha 2	1.19 ± 0.04	2.00 ± 0.47
	HBM	hemoglobin mu	0.96 ± 0.04	1.73 ± 0.16
	HBQ1	hemoglobin theta 1	1.09 ± 0.05	1.06 ± 0.12
	HBZ	hemoglobin zeta	0.31 ± 0.08	3.62 ± 1.19
	HBZP1	hemoglobin zeta pseudogene 1	---	11.56 ± 7.90
Globin switching regulators	KLF1	Kruppel-like factor 1 (erythroid)	1.02 ± 0.02	0.94 ± 0.02
	ZBTB7A	zinc finger and BTB domain containing 7A	0.98 ± 0.04	0.95 ± 0.07
	BCL11A	B-cell CLL/lymphoma 11A (zinc finger protein)	1.17 ± 0.09	0.80 ± 0.06
	PPARGC1A	peroxisome proliferator-activated receptor gamma coactivator 1 alpha	---	---
	MYB	v-myb avian myeloblastosis viral oncogene homolog	0.91 ± 0.01	0.73 ± 0.13
	IKZF1	IKAROS family zinc finger 1 (Ikaros)	1.07 ± 0.01	1.37 ± 0.16
	SOX6	SRY (sex determining region Y)-box 6	1.70 ± 0.07	1.87 ± 0.38
	TAL1	T-cell acute lymphocytic leukemia 1	1.12 ± 0.04	1.22 ± 0.12
	GATA1	GATA binding protein 1 (globin transcription factor 1)	0.97 ± 0.04	1.11 ± 0.12
	LMO2	LIM domain only 2 (rhombotin-like 1)	1.28 ± 0.04	1.84 ± 0.35
	ZFPM1	zinc finger protein FOG family member 1	0.90 ± 0.09	0.95 ± 0.17
	TCF3	transcription factor 3	0.92 ± 0.02	1.11 ± 0.20
	LIN28B	lin-28 homolog B (C. elegans)	0.91 ± 0.07	5.28 ± 2.41
	LDB1	LIM domain binding 1	1.03 ± 0.04	1.07 ± 0.08
	DNMT1	DNA (cytosine-5-)-methyltransferase 1	0.97 ± 0.02	0.91 ± 0.10
	KDM1A	lysine (K)-specific demethylase 1A	0.88 ± 0.00	0.96 ± 0.13
	CHD3	chromodomain helicase DNA binding protein 3	0.88 ± 0.02	1.05 ± 0.08
	CHD4	chromodomain helicase DNA binding protein 4	1.00 ± 0.02	1.06 ± 0.05
	MTA1	metastasis associated 1	0.92 ± 0.04	1.25 ± 0.10
	MTA2	metastasis associated 1 family member 2	0.95 ± 0.02	1.16 ± 0.04
	HDAC1	histone deacetylase 1	0.96 ± 0.03	0.93 ± 0.04
	HDAC2	histone deacetylase 2	0.95 ± 0.03	0.91 ± 0.23
	MBD2	methyl-CpG binding domain protein 2	1.03 ± 0.02	1.09 ± 0.06
	MBD3	methyl-CpG binding domain protein 3	0.96 ± 0.02	0.79 ± 0.14
	RBBP4	retinoblastoma binding protein 4	0.96 ± 0.03	0.81 ± 0.12
	RBBP7	retinoblastoma binding protein 7	0.98 ± 0.03	0.82 ± 0.02
	GATAD2A	GATA zinc finger domain containing 2A	0.96 ± 0.03	0.93 ± 0.11
	GATAD2B	GATA zinc finger domain containing 2B	1.02 ± 0.04	1.19 ± 0.12
	NR2C1	Nuclear Receptor Subfamily 2 Group C Member 1	1.11 ± 0.04	0.58 ± 0.16
	NR2C2	Nuclear Receptor Subfamily 2 Group C Member 2	0.97 ± 0.04	1.26 ± 0.26
	BGLT3	LncRNA-BGL3, Beta Globin Locus Transcript 3	3.40 ± 0.34	10.02 ± 6.54
	MIRLET7A1	microRNA let-7a-1	---	0.86 ± 1.79
	MIR16-1	microRNA 16-1	---	---
	MIR26B	microRNA 26b	0.96 ± 0.78	5.02 ± 8.73
	MIR96	microRNA 96	---	---

Supplementary Table 8-1. List of the overlapped genes in Supplementary Figure 7b and 9e. The statistical test (adjp) was determined using multiple comparisons analysis, Benjamini-Hochberg adjusted FDR test of the default of AltAnalyze (Ver. 2.0).

shG9a		UP: 27	
Symbol	Name	FoldChange	adjp
CCDC71L	coiled-coil domain containing 71 like(CCDC71L)	2.05	1.05.E-02
ULBP1	UL16 binding protein 1(ULBP1)	2.44	3.01.E-03
FRY	FRY microtubule binding protein(FRY)	2.77	4.29.E-02
RAET1K	retinoic acid early transcript 1K pseudogene(RAET1K)	2.84	3.30.E-02
IGSF3	immunoglobulin superfamily member 3(IGSF3)	2.88	5.14.E-03
MYO5B	myosin VB(MYO5B)	3.00	3.83.E-02
HCLS1	hematopoietic cell-specific Lyn substrate 1(HCLS1)	3.11	4.96.E-02
EFCAB10	EF-hand calcium binding domain 10(EFCAB10)	3.79	4.38.E-02
LTF	lactotransferrin(LTF)	5.08	4.52.E-02
HBE1	hemoglobin subunit epsilon 1(HBE1)	5.62	2.63.E-03
RUNDC3A	RUN domain containing 3A(RUNDC3A)	5.82	1.58.E-03
RP11-180K7.1		6.21	2.43.E-02
CTD-2643I7.1	beta globin locus transcript 3(BGLT3)	6.73	1.65.E-02
PRG2	proteoglycan 2, pro eosinophil major basic protein(PRG2)	7.20	1.03.E-04
LINC01033	long intergenic non-protein coding RNA 1033(LINC01033)	7.33	1.43.E-02
GS1-122H1.2		12.26	1.38.E-05
TRAF3IP3	TRAF3 interacting protein 3(TRAF3IP3)	12.78	5.15.E-03
RP11-538D16.2		15.31	2.81.E-05
NR5A2	nuclear receptor subfamily 5 group A member 2(NR5A2)	16.62	1.31.E-03
LAMB3	laminin subunit beta 3(LAMB3)	18.68	7.19.E-06
C1orf115	chromosome 1 open reading frame 115(C1orf115)	19.52	2.63.E-03
ARHGAP25	Rho GTPase activating protein 25(ARHGAP25)	19.64	7.10.E-04
PASD1	PAS domain containing repressor 1(PASD1)	19.98	4.99.E-04
RAB27B	RAB27B, member RAS oncogene family(RAB27B)	29.08	1.42.E-06
HBG2	hemoglobin subunit gamma 2(HBG2)	34.29	3.27.E-03
HBG1	hemoglobin subunit gamma 1(HBG1)	58.47	1.06.E-03
ADAMTS19	ADAM metallopeptidase with thrombospondin type 1 motif 19(ADAMTS19)	147.11	1.96.E-04

shG9a		DOWN: 12	
Symbol	Name	FoldChange	adjp
TMEM176B	transmembrane protein 176B(TMEM176B)	-12.48	1.09.E-03
TMEM176A	transmembrane protein 176A(TMEM176A)	-9.90	2.89.E-03
ZNF160	zinc finger protein 160(ZNF160)	-6.21	3.86.E-02
SPTBN2	spectrin beta, non-erythrocytic 2(SPTBN2)	-4.98	1.15.E-03
GFPT2	glutamine-fructose-6-phosphate transaminase 2(GFPT2)	-4.45	1.32.E-02
LPPR3	phospholipid phosphatase related 3(PLPPR3)	-3.88	4.40.E-03
PRSS57	serine protease 57(PRSS57)	-3.60	2.45.E-02
CFD	complement factor D(CFD)	-2.66	8.95.E-04
ZNF320	zinc finger protein 320(ZNF320)	-2.59	1.77.E-03
ZNF611	zinc finger protein 611(ZNF611)	-2.57	4.00.E-03
ZNF559	zinc finger protein 559(ZNF559)	-2.34	4.65.E-03
CTD-2006C1.2	ZNF433 and ZNF878 antisense RNA 1(ZNF433-AS1)	-2.24	1.75.E-02

Supplementary Table 8-2. List of the overlapped genes in Supplementary Figure 7b and 9e.

BCL11A KO		UP: 35	
Symbol	Name	FoldChange	adjp
SAMD9	sterile alpha motif domain containing 9(SAMD9)	2.44	1.77.E-02
CCDC71L	coiled-coil domain containing 71 like(CCDC71L)	2.57	3.35.E-03
AC006116.22		2.85	1.58.E-02
AC006116.17		2.94	2.00.E-03
AKR1C1	aldo-keto reductase family 1 member C1(AKR1C1)	3.09	1.42.E-02
TSPO2	translocator protein 2(TSPO2)	3.46	5.42.E-03
NECAB1	N-terminal EF-hand calcium binding protein 1(NECAB1)	3.56	3.82.E-02
HEPH	hephaestin(HEPH)	3.98	1.72.E-02
SYN1	synapsin I(SYN1)	4.36	9.47.E-04
PASD1	PAS domain containing repressor 1(PASD1)	4.74	3.63.E-03
CASP4	caspase 4(CASP4)	4.87	1.56.E-02
ARHGAP25	Rho GTPase activating protein 25(ARHGAP25)	5.18	6.44.E-03
RP11-284F21.10		5.25	2.81.E-02
RP11-63D14.1		6.64	1.33.E-03
ZNF365	zinc finger protein 365(ZNF365)	8.01	6.10.E-03
RHD	Rh blood group D antigen(RHD)	8.41	5.18.E-04
CD300C	CD300c molecule(CD300C)	9.44	4.73.E-02
CR1L	complement C3b/C4b receptor 1 like(CR1L)	10.13	3.62.E-04
LINC01033	long intergenic non-protein coding RNA 1033(LINC01033)	10.54	1.28.E-03
LAMB3	laminin subunit beta 3(LAMB3)	10.79	7.19.E-03
MACC1	MET transcriptional regulator MACC1(MACC1)	11.84	2.61.E-04
C12orf79	long intergenic non-protein coding RNA 1619(LINC01619)	15.06	1.35.E-02
SPRR2F	small proline rich protein 2F(SPRR2F)	18.72	1.06.E-02
KRT79	keratin 79(KRT79)	21.05	5.00.E-03
RUNX2	RUNX family transcription factor 2(RUNX2)	25.64	1.19.E-03
TRIM10	tripartite motif containing 10(TRIM10)	28.33	6.82.E-05
CTD-2643I7.1	beta globin locus transcript 3(BGLT3)	35.82	1.61.E-03
TMEM74	transmembrane protein 74(TMEM74)	37.86	2.27.E-04
PTPRC	protein tyrosine phosphatase receptor type C(PTPRC)	39.43	5.48.E-03
HBG2	hemoglobin subunit gamma 2(HBG2)	44.78	1.35.E-04
RUNDC3A	RUN domain containing 3A(RUNDC3A)	51.67	3.92.E-03
ALDH1A1	aldehyde dehydrogenase 1 family member A1(ALDH1A1)	194.70	1.73.E-03
HBG1	hemoglobin subunit gamma 1(HBG1)	244.34	2.16.E-03
HBE1	hemoglobin subunit epsilon 1(HBE1)	493.51	8.80.E-04
ABLIM3	actin binding LIM protein family member 3(ABLIM3)	1433.45	8.07.E-04

BCL11A KO		DOWN: 5	
Symbol	Name	FoldChange	adjp
PRSS57	serine protease 57(PRSS57)	-63.54	2.23.E-04
RNF125	ring finger protein 125(RNF125)	-4.82	3.34.E-03
FGFR4	fibroblast growth factor receptor 4(FGFR4)	-4.39	3.30.E-03
DYSF	dysferlin(DYSF)	-2.76	6.57.E-03
ZNF559	zinc finger protein 559(ZNF559)	-2.01	1.36.E-02

Supplementary Table 8-3. List of the overlapped genes in Supplementary Figure 7b and 9e.

ZBTB7A KO		UP: 45	
Symbol	Name	FoldChange	adjp
CCDC71L	coiled-coil domain containing 71 like(CCDC71L)	2.20	6.26.E-03
SYP	synaptophysin(SYP)	2.41	3.47.E-02
ARHGAP25	Rho GTPase activating protein 25(ARHGAP25)	2.64	2.38.E-02
TSPO2	translocator protein 2(TSPO2)	2.72	5.10.E-03
RHD	Rh blood group D antigen(RHD)	3.00	1.82.E-03
KIF4B	kinesin family member 4B(KIF4B)	3.00	6.99.E-03
TRIM10	tripartite motif containing 10(TRIM10)	3.03	4.74.E-03
AKR1C1	aldo-keto reductase family 1 member C1(AKR1C1)	3.22	7.02.E-03
EGFL6	EGF like domain multiple 6(EGFL6)	3.31	4.53.E-03
RP11-34P13.7		3.76	1.75.E-03
CD84	CD84 molecule(CD84)	4.26	2.99.E-03
PASD1	PAS domain containing repressor 1(PASD1)	4.61	2.63.E-02
FPR3	formyl peptide receptor 3(FPR3)	4.64	4.66.E-03
AC006116.22		4.75	1.76.E-03
NECAB1	N-terminal EF-hand calcium binding protein 1(NECAB1)	5.33	6.17.E-03
AC006116.17		5.63	1.92.E-04
SPRR2F	small proline rich protein 2F(SPRR2F)	5.85	4.83.E-02
IFI16	interferon gamma inducible protein 16(IFI16)	6.96	6.25.E-03
RP11-38M8.1	uncharacterized LOC105375519(LOC105375519)	7.57	3.12.E-02
SMIM14	small integral membrane protein 14(SMIM14)	10.51	2.52.E-02
ZNF365	zinc finger protein 365(ZNF365)	10.64	2.32.E-03
SYN1	synapsin I(SYN1)	11.28	2.33.E-04
PRG2	proteoglycan 2, pro eosinophil major basic protein(PRG2)	12.02	6.04.E-04
IL18RAP	interleukin 18 receptor accessory protein(IL18RAP)	14.63	4.40.E-03
TMEM74	transmembrane protein 74(TMEM74)	24.16	2.13.E-04
ALDH1A1	aldehyde dehydrogenase 1 family member A1(ALDH1A1)	24.55	5.97.E-03
LAMB3	laminin subunit beta 3(LAMB3)	31.26	1.05.E-03
HEPH	hephaestin(HEPH)	31.81	5.63.E-04
CTD-2643I7.1	beta globin locus transcript 3(BGLT3)	34.90	9.76.E-04
RUNDC3A	RUN domain containing 3A(RUNDC3A)	37.90	4.53.E-03
HBG2	hemoglobin subunit gamma 2(HBG2)	38.87	7.05.E-05
OR2AT4	olfactory receptor family 2 subfamily AT member 4(OR2AT4)	40.60	3.25.E-03
ATP1B1	ATPase Na+/K+ transporting subunit beta 1(ATP1B1)	47.90	7.76.E-03
NR5A2	nuclear receptor subfamily 5 group A member 2(NR5A2)	49.82	2.92.E-04
CPA3	carboxypeptidase A3(CPA3)	113.86	6.16.E-06
FMN1	formin 1(FMN1)	125.86	1.02.E-04
HBE1	hemoglobin subunit epsilon 1(HBE1)	126.25	1.39.E-03
CSMD2	CUB and Sushi multiple domains 2(CSMD2)	128.24	1.82.E-03
KRT79	keratin 79(KRT79)	180.42	4.35.E-04
HBG1	hemoglobin subunit gamma 1(HBG1)	188.49	1.47.E-03
MS4A2	membrane spanning 4-domains A2(MS4A2)	254.03	2.89.E-03
RAB27B	RAB27B, member RAS oncogene family(RAB27B)	307.21	2.84.E-05
RUNX2	RUNX family transcription factor 2(RUNX2)	321.89	9.56.E-05
ABLIM3	actin binding LIM protein family member 3(ABLIM3)	333.29	9.82.E-04
PTPRC	protein tyrosine phosphatase receptor type C(PTPRC)	618.70	5.08.E-04

ZBTB7A KO		DOWN: 5	
Symbol	Name	FoldChange	adjp
PRSS57	serine protease 57(PRSS57)	-8.28	6.63.E-04
LPPR3	phospholipid phosphatase related 3(PLPPR3)	-3.70	3.76.E-03
ZNF772	zinc finger protein 772(ZNF772)	-3.68	7.23.E-03
FGFR4	fibroblast growth factor receptor 4(FGFR4)	-2.99	1.53.E-03
DYSF	dysferlin(DYSF)	-2.62	2.02.E-03

Supplementary Table 9. Small molecule screening data.

Category	Parameter	Description
Assay	Type of assay	*In vitro* fluorogenic assay
	Target	G9a
	Primary measurement	Detection of the fluorescence intensity at λex 330 nm/λem 380 nm
	Key reagents	A recombinant mG9a protein, peptidyl-MCA substrate, and SAM
	Assay protocol	The section in the methods
	Additional comments	None
Library	Library size	141,117 compounds
	Library composition	Synthetic compounds with structural diversity
	Source	Drug Discovery Initiative at The University of Tokyo (Tokyo, Japan)
	Additional comments	None
Screen	Format	384-well plates
	Concentration(s) tested	4 or 20 μM, 1% DMSO
	Plate controls	High control: DMSO without SAM, Low control: DMSO
	Reagent/ compound dispensing system	EDR-384UX (BioTec, Tokyo, Japan)<br>LD-02 single line dispenser (BioTec, Tokyo, Japan)
	Detection instrument and software	Synergy H4 (BioTek, Winooski, USA)<br>Genedata Screener (Genedata, Basel, Switzerland)
	Assay validation/QC	Z'-factor was used for QC
	Correction factors	No specific correction was used
	Normalization	Inhibition %=100 x (Mean of low control – Sample well)/(Mean of low control – Mean of high control)
	Additional comments	None
Post-HTS analysis	Hit criteria	Inhibition ≥ 30% at 20 μM<br>Inhibition ≥ 15% at 4 μM
	Hit rate	0.07%
	Additional assay(s)	AlphaLISA-based assay
	Confirmation of hit purity and structure	Compounds were repurchased (Enamine, Kiev, Ukraine) and resynthesized.
	Additional comments	None

Supplementary Table 10. Oligonucleotide primers used for quantitative PCR.

		Sequence (5'-3')
ChIP-qPCR	HS4_F	TCTCCCACTCAGCAGCTATGAG
	HS4_R	TGCTGGGCCTTGGAGTGA
	HS3_F	GGGCTGGCCACCAGCTAT
	HS3_R	CACCAACCTGACCTAACCACCTA
	HS2_F	GGCTCAAGCACAGCAATGC
	HS2_R	CATCACTCTAGGCTGAGAACATCTG
	HS1_F	GCAATAGCAGGAGCATTCTGACT
	HS1_R	GGACTATGCTGAGCTGTGATG
	γ pro_F	CAAATATCTGTCTGAAACGGTCCCT
	γ pro_R	TGCCTTGTCAAGGCTATTGGT
	δ pro_F	AACCCTGCTTATCTTAAACCAACCT
	δ pro_R	CTCTGCCCTGCCTTTTATGC
	β pro_F	GAGGGTTTGAAGTCCAACTCCTAA
	β pro_R	CAGGGTGAGGTCTAAGTGATGACA
ChIP-qPCR & RT-qPCR	BGLT3_F	AAGATAATCTTGGTTTTGCCTCAAA
	BGLT3_R	TCTACTTGATATAGTTGAGAGGCAGTTACC
RT-qPCR	HBB_F	CAGTGCAGGCTGCCTATC
	HBB_R	ATACTTGTGGGCCAGGGCAT
	HBG1/2_F	TGGATGATCTCAAGGGCAC
	HBG1/2_R	TCAGTGGTATCTGGAGGACA
	BCL11A_F	ACAAACGGAAACAATGCAATGG
	BCL11A_R	TTTCATCTCGATTGGTGAAGGG
	ZBTB7A_F	GCTTGGGCCGGTTGAATGTA
	ZBTB7A_R	GGCTGTGAAGTTACCGTCGG
	GAPDH_F	GCACCGTCAAGGCTGAGAAC
	GAPDH_R	TGGTGAAGACGCCAGTGGA
	mouse Hba-α1_F	TCACTTTGATGTAAGCCACGG
	mouse Hba-α1_R	TCAGAGCAGACAAGGCACC
	mouse Hbb-εy_F	ACCCTCATCAATGGCCTGTG
	mouse Hbb-εy_R	TGGCAGAAGCAGAGGACAAG
	mouse Hbb-βh1_F	TGGATCCTGAGAACTTCAAGC
	mouse Hbb-βh1_R	ATTGGCCACTCCAATCACC
	mouse β(maj/min)_F	TTTAACGATGGCCTGAATCACTT
	mouse β(maj/min)_R	CAGCACAATCACGATCATATTGC
	mouse Gapdh_F	TGGTGAAGGTCGGTGTGAAC
	mouse Gapdh_R	CCATGTAGTTGAGGTCAATGAAGG

Supplementary Table 11. Oligonucleotide primers used for construction of plasmids.

vector	gene	Sequence (5'-3')
pCX4-pur	BGLT3_F	AGTGTGGTGGTACGGGAATTCCCACAAAGGGTTTATATTGAGG
	BGLT3_R	TTAACAATTGTTACCGCGGCCGCTAAGGGGAAAACTGGGTTTTATTAC
pIRES-3xFLAG	BCL11A_F	ATGACGATGACAAGGAATTCATGTCTCGCCGCAAGCAAG
	BCL11A_R	CCCTCGAGCGGCCGCTCTAGATCAGAACTTAAGGGCTCTCG
	ZBTB7A_F	ATGACGATGACAAGGAATTCATGGCCGGCGGCGTGGAC
	ZBTB7A_R	CCCTCGAGCGGCCGCTCTAGATTAGGCGAGTCCGGCTGTG
pcDNA3.1(-)/myc-his B	CHD3_F	AACGGGCCCTCTAGACTCGAGGCCACCATGGATAAGGATGACATTCGGCTG
	CHD3_R	TAGTCCAGTGTGGTGGAATTCGTTCAGAACCTTGTGCAGGAC
	CHD4_F	AACGGGCCCTCTAGACTCGAGGCCACCATGGCGTCGGGCCTGGG
	CHD4_R	TAGTCCAGTGTGGTGGAATTCCTGCTGCTGGGCTACCTG
pSUPER	non-targeting_F	GATCCCCAAATCACAGAATCGTCGTATTCAAGAGATACGACGATTCTGTGATTTTTTTA
	non-targeting_R	AGCTTAAAAAAATCACAGAATCGTCGTATCTCTTGAATACGACGATTCTGTGATTTGGG
	G9a_F	GATCCCCCTACTCTGTGGATGAGCGCTTCAAGAGAGCGCTCATCCACAGAGTAGTTTTTA
	G9a_R	AGCTTAAAAACTACTCTGTGGATGAGCGCTCTCTTGAAGCGCTCATCCACAGAGTAGGGG
	BGLT3_F	GATCCCCCATCGATCTCTGCTCTTAATTCAAGAGATTAAGAGCAGAGATCGATGTTTTTA
	BGLT3_R	AGCTTAAAAACATCGATCTCTGCTCTTAATCTCTTGAATTAAGAGCAGAGATCGATGGGG

Supplementary Table 12. MRM transitions for target peptides.

protein	peptide	precursor m/z	product m/z	collision energy (eV)
Hemoglobin subunit gamma-1	LHVDPENFK	549.8	251.2 (b2), 634.3 (y5), 749.3 (y6)	22
	LHVD[P*]ENFK	552.8	251.2 (b2), 640.3 (y5), 755.4 (y6)	22
Hemoglobin subunit beta	LHVDPENFR	563.8	251.2 (b2), 662.3 (y5), 777.4 (y6)	23
	LHVD[P*]ENFR	566.8	251.2 (b2), 668.3 (y5), 783.4 (y6)	23
Hemoglobin subunit alpha	FLASVSTVLTSK	626.9	735.4 (y7), 921.5 (y9), 992.6 (y10)	25
	FLASVSTV[L*]TSK	630.4	742.4 (y7), 928.5 (y9), 999.6 (y10)	25

[P*] and [L*] denote isotope-labeled amino acids.

Supplementary Note

Compound synthesis.

General Procedure. All chemicals were purchased from commercial suppliers TCI, Wako, and Sigma-Aldrich, and used as received unless otherwise specified. Melting points were measured using an OptiMelt MPA100 automated melting point system. Infrared (IR) spectra were recorded with a PerkinElmer Spectrum 100 spectrometer. Measurements of MS and high-resolution mass spectrometry (HRMS) were performed with a JEOL JMS-T100LP or Synapt G2 instrument (Waters) or Q Exactive (Thermo Fisher Scientific) mass spectrometer. Proton nuclear magnetic resonance (^{1}H NMR) spectra were measured with a JEOL JMN-ECA400 (400 MHz) or JEOL JMN-ECX400P (400 MHz) spectrometer. The chemical shifts are expressed in parts per million (δ value) downfield from tetramethylsilane, using tetramethylsilane (δ = 0) and/or residual solvents such as chloroform (δ = 7.26) and dimethyl sulfoxide (δ = 2.50) as an internal standard. Splitting patterns are indicated as follows: s, singlet; d, doublet; t, triplet; q, quartet; m, multiplet; br; broad peak. Purity data were collected by an Agilent 1100 HPLC or U3000 (Thermo Fisher Scientific) with diode array detector. The column used was an RP-AQUA (50 mm × 2.1 mm ID, 2.6 μm; ChromaNik Technologies). The method is as follows: ESI+, flux of 0.5 mL/min, 5−95% MeCN in H_2O + 0.05% formic acid, total run time of 10 min (λ = 190–400 nm). Column chromatography was carried out with silica gel 60 (Kanto Chemical) as an absorbent. Merck pre-coated thin-layer chromatography (TLC) plates (silica gel 60 F254, 0.25 mm, Art 5715) were used for the TLC analysis. All purities of final compounds were ≥95.0% and were measured using HPLC.

Compound synthesis in Supplementary Fig. 15.

Methyl (S)-2-(3-(methylthio)-1-(3,6,6-trimethyl-4-oxo-4,5,6,7-tetrahydro-1H-indole-2-carboxamido)propyl)-1H-benzo[d]imidazole-6-carboxylate (9).

a) To a solution of Boc-Met-OH (1.0 g, 4.01 mmol), methyl 3,4-diaminobenzoate (733 mg, 4.41 mmol) and HOBt (150 mg, 4.81 mmol) in DMF (20 mL) were added DIEA (1.05 mL, 4.81 mmol) and EDCI-HCl (923 mg, 4.81 mmol) at 0 ° C under nitrogen atmosphere. The reaction mixture was stirred overnight at ambient temperature before being quenched with water. The aqueous layer was separated and extracted with ethyl acetate. Combined organic layer was washed with water and brine, dried over anhydrous sodium sulfate, and concentrated under reduced pressure after filtration. The residue was dissolved in acetic acid (12 mL) at ambient temperature under nitrogen atmosphere. The mixture was stirred at 60 ° C for 1 h, and then evaporated under reduced pressure before being quenched with saturated aqueous sodium bicarbonate. The aqueous layer was separated and extracted with ethyl acetate. Combined organic layer was washed with brine, dried over anhydrous sodium sulfate and concentrated under reduced pressure after filtration. The residue was purified by column chromatography on silica gel (ethyl acetate–hexane) to give methyl (S)-2-(1-((tert-butoxycarbonyl)amino)-3-(methylthio)propyl)-1H-benzo[d]imidazole-5-carboxylate (11.38 g, 91.0%) as a pale-red amorphous. LC–MS (ESI) m/z: 380 (M + H^+). ^{1}H NMR (500 MHz, $CDCl_3$): 1.45 (9H, s), 2.09 (3H, s), 2.26–2.39 (1H, m), 2.43–2.55 (1H, m), 2.57–2.71 (2H, m), 3.94 (3H, s), 5.03–5.14 (1H, m), 5.42 (1H, s), 7.37–7.77 (1H, m), 7.97 (1H, m), 8.10–8.49 (1H, m), 10.57 (1H, brs).

b) To a solution of the above compound (1.6 g, 4.2 mmol) in ethyl acetate (10 mL) was added a solution of 4 M hydrogen chloride in ethyl acetate (10.5 mL, 42 mmol) at ambient temperature under nitrogen atmosphere. The reaction mixture was stirred for 2 h at ambient temperature and then evaporated. Precipitate was washed with IPE, dried under reduced pressure to afford methyl (S)-2-(1-amino-3-(methylthio)propyl)-1H-benzo[d]imidazole-5-carboxylate hydrochloride (1.38 g, quant.) as a powder. LC–MS (ESI) m/z: 280 (M + H^+). ^{1}H NMR (500 MHz, $CDCl_3$–CD_3OD): 2.05 (3H, s), 2.55–2.65 (2H, m), 2.70–2.85 (2H, m), 3.99 (3H, s), 5.45 (1H, m), 7.88 (1H, d, J= 8.8 Hz), 8.24 (1H, d, J= 8.8 Hz), 8.50 (1H, s).

c) To a solution of the above compound (139 mg, 0.54 mmol), 3,6,6-trimethyl-4-oxo-4,5,6,7-tetrahydro-1H-indole-2-carboxylic acid (50 mg, 0.23 mmol) and HOBt (36.5 mg, 0.27 mmol) in DMF (5 mL) were added DIEA (0.047 mL, 0.27 mmol) and EDCI-HCl (36.5 mg, 0.27 mmol) at 0 ° C under nitrogen atmosphere. The reaction mixture was stirred overnight at ambient temperature before being quenched with water. The aqueous layer was separated and extracted with ethyl acetate. Combined organic layer was washed with water and brine, dried over anhydrous sodium sulfate, and concentrated under reduced pressure after filtration. The residue was purified by flash column chromatography on silica gel (ethyl acetate–hexane) and triturated with IPE to afford compound **9** (30 mg, 27.6%) as a colorless powder. LC–MS (ESI) m/z: 483 (M + H^+). ^{1}H NMR (500 MHz, $CDCl_3$): δ [ppm] 1.09 (6H, s), 2.14 (3H, s), 2.10–2.22 (1H, m), 2.24–2.33 (1H, m), 2.34 (2H, s), 2.58–2.78 (2H, m), 2.64 (2H, s), 2.69 (3H, s), 4.92 (1H, q, J= 7.1 Hz), 6.77 (1H, d, J= 7.7 Hz), 7.12 (1H, t, J= 7.4 Hz), 7.32 (2H, t, J= 7.6 Hz), 7.53 (2H, d, J= 8.1 Hz), 8.52 (1H, s), 9.39 (1H, brs).

Compounds **6** and **8** were synthesized following the same procedure of compound **9**.

(S)-N-(1-(1H-benzo[d]imidazol-2-yl)-3-(methylthio)propyl)-3,6,6-trimethyl-4-oxo-4,5,6,7-tetrahydro-1H-indole-2-carboxamide (**6**). LC–MS (ESI) m/z: 425 (M + H$^+$). HRMS m/z: (M + H$^+$) observed for $C_{14}H_{24}N_2O_2$ + H$^+$: 253.19160; found, 253.19083. ^{1}H NMR (500 MHz, DMSO-d$_6$): δ [ppm] 1.02 (s, 6H), 2.08 (s, 3H), 2.15–2.28 (m, 3H), 2.30–2.41 (m, 1H), 2.49 (s, 3H), 2.55–2.63 (m, 2H), 2.65 (s, 2H), 5.38–5.44 (m, 1H), 7.11–7.21 (m, 2H), 7.38–7.64 (m, 2H), 7.97 (d, J= 8.2 Hz, 1H), 11.60 (s, 1H), 12.33 (s, 1H).

(S)-N-(1-(1H-benzo[d]imidazol-2-yl)-3-(methylthio)propyl)-4,5-dihydro-2H-benzo[g]indazole-3-carboxamide (8). LC–MS (ESI) m/z: 418 (M + H$^+$). ^{1}H NMR (500 MHz, $CDCl_3$): δ [ppm] 1.98 (3H, s), 2.40–2.72 (4H, m), 2.87–3.10 (3H, m), 3.16–3.28 (1H, m), 5.78–5.93 (1H, m), 7.15–7.32 (6H, m), 7.34–7.47 (1H, m), 7.69–7.92 (2H, m),10.00 (1H, s), 11.05 (1H, s).

(S)-3,6,6-trimethyl-N-(4-(methylthio)-1-oxo-1-(phenylamino)butan-2-yl)-4-oxo-4,5,6,7-tetrahydro-1H-indole-2-carboxamide (10).
The above-mentioned compound was obtained following the same procedure b and c as a colorless powder.
LC–MS (ESI) m/z: 428 (M + H$^+$). ^{1}H NMR (500 MHz, $CDCl_3$): δ [ppm] 1.06 (6H, d, J= 13.0 Hz), 2.05 (3H, s), 2.32 (2H, s), 2.40–2.50 (1H, m), 2.53–2.75 (3H, m), 2.58 (2H, s), 2.65 (3H, s), 3.93 (3H, s), 5.54–5.63 (1H, m), 6.95–7.13 (1H, m), 7.34–7.76 (1H, m), 7.95 (1H, d, J=8.8 Hz), 8.08–8.47 (1H, m), 9.70 (1H, s), 11.11 (1H, s).

Compound synthesis in Supplementary Fig. 16.

The synthesis of **11** (RK-701) and *R*-**11** (RK-0133114) is outlined in Supplementary Fig. 16. Commencing with commercially available **1**, an alkylation reaction with **2** afforded compound **3**, and the *N*-protected amino acid ester *rac*-**5** was synthesized in a two-step procedure via hydrolysis under basic conditions and decarboxylation reaction. *rac*-**8** was obtained by removal of the Boc group followed by condensation with carboxylic acid **7** under standard conditions. The ester moiety of *rac*-**8** was hydrolyzed under basic conditions, and condensation of the resultant carboxylic acid *rac*-**9** with 4-cyanoaniline (**10**) resulted in *rac*-**11.** Furthermore, the synthesis of the desired **11** (RK-701) and *R*-**11** (RK-0133114) was accomplished by optical resolution of *rac*-**11** using chiral column chromatography.

diethyl 2-((*tert*-butoxycarbonyl)amino)-2-(2-cyclopropylethyl)malonate **(3).** To a solution of **2** (0.796 mL, 3.12 mmol) in DMF (5.20 mL) was added potassium *tert*-butoxide (327 mg, 2.91 mmol) at ambient temperature under an argon atmosphere. The reaction mixture was stirred for 30 min at 70° C before being cooled to ambient temperature. The resultant reaction mixture was added a solution of **1** (500 mg, 2.08 mmol) in DMF (5.20 mL) at ambient temperature under an argon atmosphere. The reaction mixture was stirred for 5 h at 70° C before being quenched with water. The organic materials were extracted with ethyl acetate and washed with water and brine. The organic phase was dried over anhydrous sodium sulfate and concentrated under reduced pressure after filtration. The residue was purified by flash column chromatography on silica gel (ethyl acetate/hexane = 10/90) to afford **3** (450 mg, 1.31 mmol, 63%) as a colorless oil. LC−MS (FI): *m/z* 343 [M]$^+$. HRMS calculated for $C_{17}H_{29}NO_6$ [M]$^+$: 343.19949; found, 343.19898. ^{1}H NMR (400 MHz, $CDCl_3$) δ −0.05–0.00 (m, 2H), 0.38–0.44 (m, 2H), 0.60–0.71 (m, 1H), 1.03–1.10 (m, 2H), 1.20–1.30 (m, 6H), 1.43 (s, 9H), 2.36–2.44 (m, 2H), 4.15–4.35 (m, 4H), 5.89 (br s, 1H).

2-((*tert*-butoxycarbonyl)amino)-4-cyclopropyl-2-(ethoxycarbonyl)butanoic acid **(*rac*-4).** To a solution of **3** (450 mg, 1.31 mmol) in EtOH (6.55 mL) was added an aqueous solution of potassium hydroxide (0.720 mL, 1.44 mmol, 2 mol/L) at ambient temperature under an argon atmosphere. The reaction mixture was stirred for 5 h at the same temperature before being concentrated under reduced pressure. The residue was acidified to pH 2 by the addition of 1 mol/L hydrochloric acid. The organic materials were extracted with ethyl acetate and washed with brine. The organic phase was dried over anhydrous sodium sulfate and concentrated under reduced pressure after filtration to afford *rac*-**4** (381 mg, 1.21 mmol, 92%) as a colorless oil. LC−MS (ESI−): *m/z* 314 [M − H]$^+$. HRMS calculated for $C_{15}H_{24}NO_6$ [M − H]$^+$: 314.16036; found, 314.15966. ^{1}H NMR (400 MHz, $CDCl_3$) δ −0.01–0.03 (m, 2H), 0.40–0.45 (m, 2H), 0.61–0.70 (m, 1H), 1.08–1.20 (m, 2H), 1.30 (t, *J* = 7.3 Hz, 3H), 1.43 (s, 9H), 2.30–2.36 (m, 2H), 4.20–4.35 (m, 2H), 5.75 (br s, 1H).

ethyl 2-((*tert*-butoxycarbonyl)amino)-4-cyclopropylbutanoate **(*rac*-5).** A solution of *rac*-**4** (381 mg, 1.21 mmol) in 1,2-dichlorobenzene (2.42 mL) was stirred for 2 h at 110° C under an argon atmosphere before being cooled to ambient temperature. The resultant reaction mixture was purified by flash column chromatography on silica gel (hexane to ethyl acetate/hexane = 20/80) to afford *rac*-**5** (281 mg, 1.04 mmol, 86%) as a colorless oil. LC−MS (ESI+): *m/z* 272 [M + H]$^+$. HRMS calculated for $C_{14}H_{26}NO_4$ [M + H]$^+$: 272.18618; found, 272.18690. ^{1}H NMR (400 MHz, $CDCl_3$) δ −0.02–0.04 (m, 2H), 0.40–0.45 (m, 2H), 0.61–0.72 (m, 1H), 1.20–1.30 (m, 5H), 1.44 (s, 9H), 1.67–1.77 (m, 1H), 1.86–1.96 (m, 1H), 4.16–4.23 (m, 2H), 4.26–4.33 (m, 1H), 4.98 (d, *J* = 7.3 Hz, 1H).

ethyl 4-cyclopropyl-2-(3,6,6-trimethyl-4-oxo-4,5,6,7-tetrahydro-1H-indole-2-carboxamido)butanoate (***rac*-8**). To a solution of *rac*-**5** (1.07 g, 3.94 mmol) in CH_2Cl_2 (9.86 mL) was added trifluoroacetic acid (9.86 mL) at ambient temperature under an argon atmosphere. The reaction mixture was stirred for 30 min at the same temperature before being concentrated under reduced pressure. To a solution of crude materials (*rac*-**6**) in DMF (19.7 mL) were added **7** (872 mg, 3.94 mmol), HOBt-H_2O (724 mg, 4.73 mmol), DIEA (2.69 mL, 15.8 mmol) and EDCI-HCl (832 mg, 4.34 mmol) at ambient temperature under an argon atmosphere. The reaction mixture was stirred for 17 h at the same temperature before being quenched with water. The organic materials were extracted with ethyl acetate and washed with brine. The organic phase was dried over anhydrous sodium sulfate and concentrated under reduced pressure after filtration. The residue was purified by flash column chromatography on silica gel (ethyl acetate/hexane = 50/50) to afford *rac*-**8** (1.29 g, 3.43 mmol, 87%) as a pale yellow powder. LC−MS (FD): *m/z* 374 $[M]^+$. HRMS calculated for $C_{21}H_{30}N_2O_4$ $[M]^+$: 374.22056; found, 374.22088. ^1H NMR (400 MHz, $CDCl_3$) δ 0.00–0.04 (m, 2H), 0.41–0.46 (m, 2H), 0.63–0.73 (m, 1H), 1.10 (s, 6H), 1.20–1.28 (m, 1H), 1.31 (t, J = 7.3 Hz, 3H), 1.32–1.38 (m, 1H), 1.84–1.95 (m, 1H), 2.02–2.13 (m, 1H), 2.34 (s, 2H), 2.64 (s, 2H), 2.67 (s, 3H), 4.20–4.28 (m, 2H), 4.79 (td, J = 7.3, 5.4 Hz, 1H), 6.38 (d, J = 7.3 Hz, 1H), 9.40 (br s, 1H).

4-cyclopropyl-2-(3,6,6-trimethyl-4-oxo-4,5,6,7-tetrahydro-1H-indole-2-carboxamido)butanoic acid (***rac*-9**). To a solution of *rac*-**8** (1.29 g, 3.44 mmol) in EtOH/THF/H_2O (34.5 mL, 1/1/1) was added lithium hydroxide monohydrate (217 mg, 5.17 mmol) at 0° C under an argon atmosphere. The reaction mixture was stirred for 6 h at ambient temperature before being concentrated under reduced pressure. The residue was acidified to pH 2 by the addition of 1 mol/L hydrochloric acid. The resultant precipitate was filtered and rinsed with water to afford *rac*-**9** (1.17 g, 3.38 mmol, 98%) as a colorless powder. LC−MS (ESI+): *m/z* 347 $[M + H]^+$. HRMS calculated for $C_{19}H_{27}N_2O_4$ $[M + H]^+$: 347.19708; found, 347.19652. ^1H NMR (400 MHz, DMSO-d_6) δ −0.05–0.00 (m, 2H), 0.32–0.37 (m, 2H), 0.59–0.69 (m, 1H), 1.01 (s, 6H), 1.13–1.31 (m, 2H), 1.69–1.80 (m, 1H), 1.81–1.91 (m, 1H), 2.20 (s, 2H), 2.46 (s, 3H), 2.63 (s, 2H), 4.24 (q, J = 7.3 Hz, 1H), 7.71 (br s, 1H).

N-(1-((4-cyanophenyl)amino)-4-cyclopropyl-1-oxobutan-2-yl)-3,6,6-trimethyl-4-oxo-4,5,6,7-tetrahydro-1*H*-indole-2-carboxamide (***rac*-11**). To a solution of *rac*-**9** (50.0 mg, 0.144 mmol) in DMF (0.722 mL) were added **10** (17.0 mg, 0.144 mmol) and DMT-MM nH_2O (47.9 mg, 0.173 mmol) at ambient temperature under an argon atmosphere. The reaction mixture was stirred for 7 h at the same temperature before being quenched with water. The organic materials were extracted with ethyl acetate and washed with brine. The organic phase was dried over anhydrous sodium sulfate and concentrated under reduced pressure after filtration. The residue was purified by flash column chromatography on silica gel (ethyl acetate/hexane = 50/50) and triturated with IPE to afford *rac*-**11** (11.9 mg, 0.0266 mmol, 19%) as a colorless powder. Mp: 224–225° C. IR (ATR) ν_{max}: 3264, 2225, 1596, 1510, 1310, 1177, 1068, 839, 548 cm^{-1}. LC−MS (ESI+): *m/z* 447 $[M + H]^+$. Retention time: 4.81 min. LC purity: >99%. HRMS calculated for $C_{26}H_{31}N_4O_3$ $[M + H]^+$: 447.23961; found, 447.24011. ^1H NMR (400 MHz, DMSO-d_6) δ 0.01–0.05 (m, 2H), 0.37–0.42 (m, 2H), 0.68–0.75 (m, 1H), 1.02 (s, 6H), 1.22–1.36 (m, 2H), 1.79–1.93 (m, 2H), 2.22 (s, 2H), 2.46 (s, 3H), 2.64 (s, 2H), 4.57 (td, J = 7.9, 5.5 Hz, 1H), 7.71 (d, J = 7.9 Hz, 1H), 7.78 (d, J = 9.2 Hz, 2H), 7.81 (d, J = 9.2 Hz, 2H), 10.58 (s, 1H), 11.65 (s, 1H).

(*S*)-*N*-(1-((4-cyanophenyl)amino)-4-cyclopropyl-1-oxobutan-2-yl)-3,6,6-trimethyl-4-oxo-4,5,6,7-tetrahydro-1*H*-indole-2-carboxamide (**11**). The title compound was separated from *rac*-**11** (15.0 mg) with CHIRALPAK IA column (EtOH/hexane = 50/50, 1.0 mL/min, 25° C) as a colorless powder (6.20 mg, 41.3%). Enantiomeric excess was determined by the same column conditions, minor enantiomer t_R = 5.78 min (0.3%), major enantiomer t_R = 7.45 min (99.7%): 99.4% *ee*.

(*R*)-*N*-(1-((4-cyanophenyl)amino)-4-cyclopropyl-1-oxobutan-2-yl)-3,6,6-trimethyl-4-oxo-4,5,6,7-tetrahydro-1*H*-indole-2-carboxamide (***R*-11**). The title compound was separated from *rac*-**11** (15.0 mg) with CHIRALPAK IA column (EtOH/hexane = 50/50, 1.0 mL/min, 25° C) as a colorless powder (5.64 mg, 37.6%). Enantiomeric excess was determined by the same column conditions, major enantiomer t_R = 5.76 min (100%), minor enantiomer t_R = 7.45 min (Not detected): 100% *ee*.

Abbreviations. DIEA, *N,N*-diisopropylethylamine; DMF, *N,N*-dimethylformamide; DMT-MM, 4-(4,6-dimethoxy-1,3,5-triazin-2-yl)-4-methylmorpholinium chloride; EDCI, 1-ethyl-3-(3-dimethylaminopropyl) carbodiimide; HOBt, 1,2,3-benzotriazol-1-ol; IPE, isopropyl ether; THF, tetrahydrofuran.

Chiral separation of R- and S-enantiomers by HPLC

Optical resolution of rac-11 by chiral column chromatography.

Column: CHIRALPACK IA (4.6 mm I.D. x 250 mm L)

Particle Size: 5 μm

Eluent: EtOH / Hexane = 50/50 < v/v >

Flow Rate: 1.0 mL/min

Column oven temperature: 25 ° C

Detector: 254 nm (UV)

Injections: 20 μL x 12, 30 μL x 26 (10 mg/mL in EtOH / Hexane (50 / 50))

Fractions were collected.

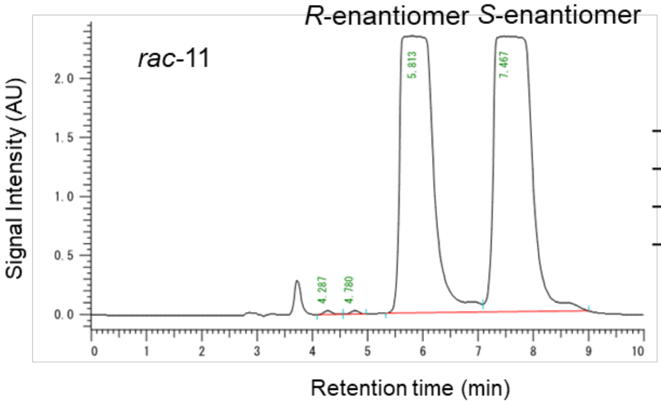

#	Compound	Time (min)	Area	Area%
1	*R*-11	5.81	47770357	47
2	11 (*S*)	7.47	53936445	53

HPLC charts of R-11 and 11.

HPLC analyses of 11 (RK-701) and R-11.

Column: CHIRALPACK IA (4.6 mm I.D. x 250 mm L)

Particle Size: 5 μm

Eluent: EtOH / Hexane = 50/50 < v/v >

Flow Rate: 1.0 mL/min

Column oven temperature: 40 ° C

Detector: 254 nm (UV)

Injection: 10 μL (10 mM in EtOH / Hexane (50 / 50))

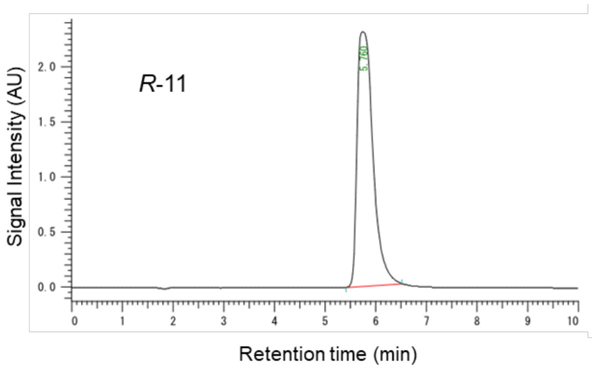

#	Compound	Time (min)	Area	Area %
1	*R*-11	5.76	2458640	100
2	11 (*S*)	-	-	-

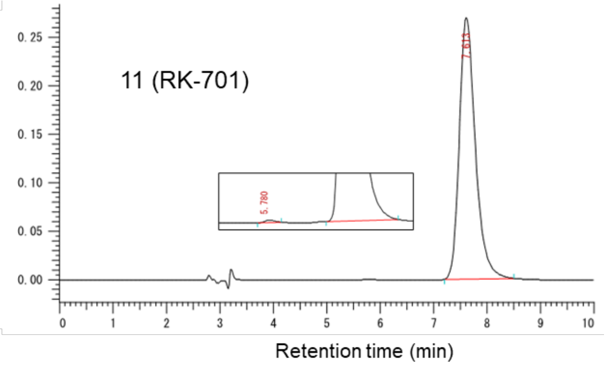

#	Compound	Time (min)	Area	Area %
1	*R*-11	5.78	138387	0.3
2	11 (*S*)	7.45	41201917	99.7

LC-MS data of RK-701

The method was performed on an Agilent 1100 HPLC with diode array detector equipped with a RP-AQUA (2.1 mm × 50 mm ID, 2.6 μm). The method was as follows: ESI+, flux of 0.5 mL/min, 5−95% MeCN in H2O + 0.05% formic acid, total run time of 9 min (λ = 190–400 nm).

LC−MS (ESI+): m/z 447 [M + H]+. Retention time: 5.59 min. LC purity: 97%.

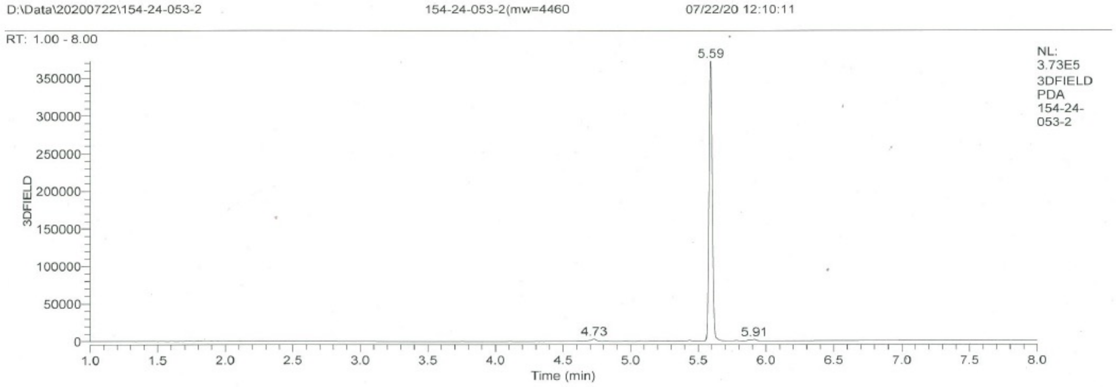

PEAK LIST
154-24-053-2.raw
RT: 0.00 - 9.00
Number of detected peaks: 7

Apex RT	Start RT	End RT	Area	%Area	Height	%Height	
4.73	4.66	4.79	8264.60	1.20	3206.20	0.84	323
5.19	5.16	5.23	706.30	0.10	420.00	0.11	
5.43	5.40	5.49	2021.30	0.29	1096.21	0.29	
5.59	5.54	5.74	670978.30	97.18	373343.43	97.63	
5.78	5.74	5.83	1632.80	0.24	685.89	0.18	
5.88	5.83	5.89	2622.00	0.38	1508.79	0.39	
5.91	5.89	5.97	4201.10	0.61	2162.44	0.57	

Gating strategy FACS analysis in Supplementary Fig. 5h.

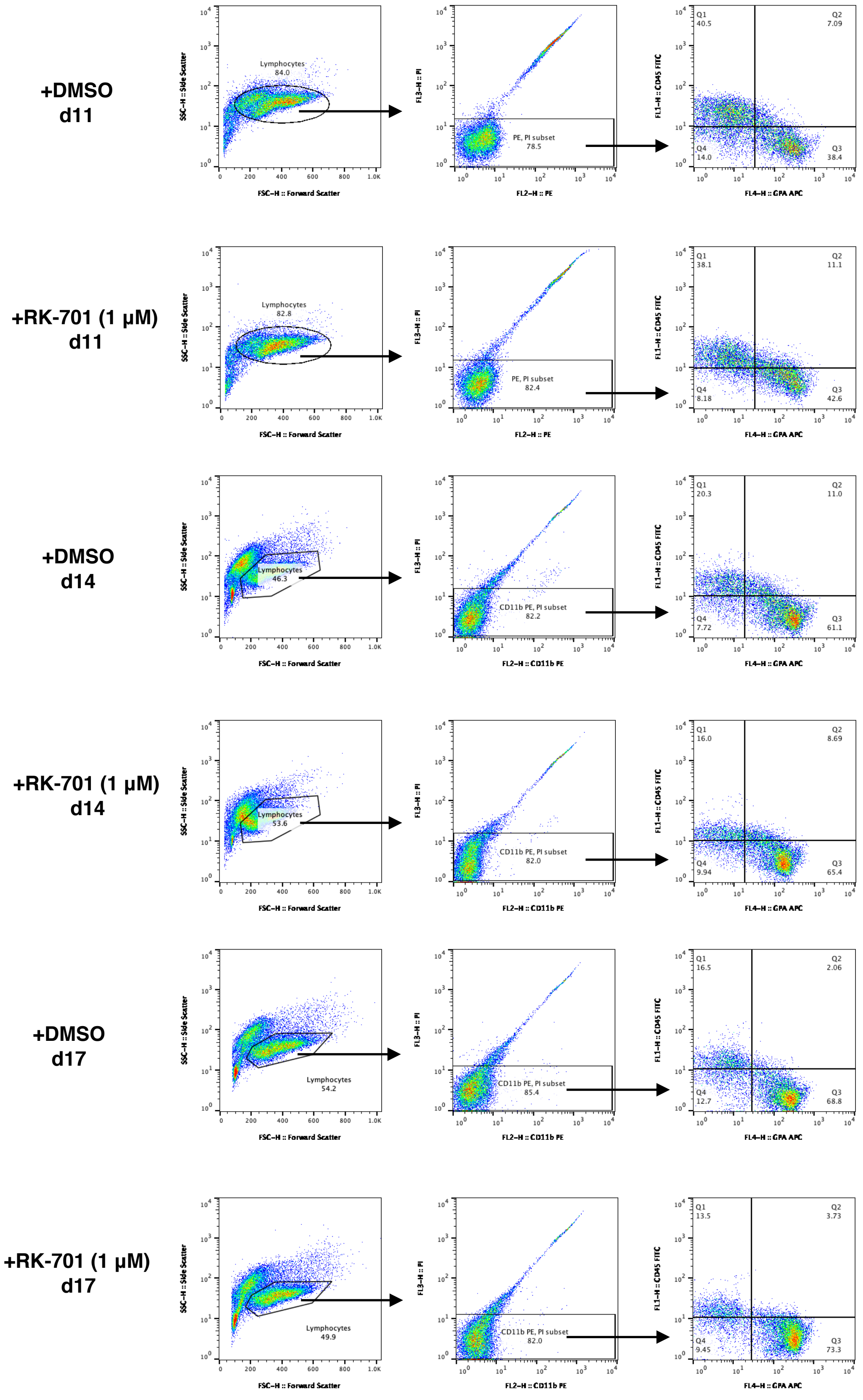

Supplementary references

1. Young, M. D., Wakefield, M. J., Smyth, G. K. & Oshlack, A. Gene ontology analysis for RNA-seq: accounting for selection bias. *Genome Biology* **11**, R14 (2010).
2. Guo, G. et al. A long noncoding RNA critically regulates Bcr-Abl-mediated cellular transformation by acting as a competitive endogenous RNA. *Oncogene* **34**, 1768–1779 (2015).
3. Ivaldi, M. S. et al. Fetal γ-globin genes are regulated by the BGLT3 long noncoding RNA locus. *Blood* **132**, 1963–1973 (2018).
4. Orkin, S. H. & Bauer, D. E. Emerging Genetic Therapy for Sickle Cell Disease. *Annual Review of Medicine* **70**, 257–271 (2019).
5. Masuda, T. et al. Transcription factors LRF and BCL11A independently repress expression of fetal hemoglobin. *Science* **351**, 285–289 (2016).
6. Johnson, R. M. et al. Fetal globin expression in New World monkeys. *J Biol Chem* **271**, 14684–14691 (1996).
7. Tan, Y., Yoder, A. D., Yamashita, N. & Li, W.-H. Evidence from opsin genes rejects nocturnality in ancestral primates. *Proceedings of the National Academy of Sciences* **102**, 14712–14716 (2005).
8. Hardison, R. C. Evolution of hemoglobin and its genes. *Cold Spring Harb Perspect Med* **2**, a011627 (2012).
