# Supplementary material for: A specific G9a inhibitor unveils BGLT3 lncRNA as a universal mediator of chemically induced fetal globin gene expression
Source: Nat Commun. 2023 Jan 12;14:23. doi: 10.1038/s41467-022-35404-0 (PMC9837035; doi:10.1038/s41467-022-35404-0)
Supplement: Supplementary file 3 — Reporting Summary [file 41467_2022_35404_MOESM3_ESM.pdf]

nature portfolio

Corresponding author(s): Minoru Yoshida
Akihiro Ito

Last updated by author(s): Nov 16, 2022

Reporting Summary

Nature Portfolio wishes to improve the reproducibility of the work that we publish. This form provides structure for consistency and transparency in reporting. For further information on Nature Portfolio policies, see our [Editorial Policies] and the [Editorial Policy Checklist].

Statistics

For all statistical analyses, confirm that the following items are present in the figure legend, table legend, main text, or Methods section.

n/a	Confirmed	
☐	☒	The exact sample size (*n*) for each experimental group/condition, given as a discrete number and unit of measurement
☐	☒	A statement on whether measurements were taken from distinct samples or whether the same sample was measured repeatedly
☐	☒	The statistical test(s) used AND whether they are one- or two-sided<br>*Only common tests should be described solely by name; describe more complex techniques in the Methods section.*
☒	☐	A description of all covariates tested
☐	☒	A description of any assumptions or corrections, such as tests of normality and adjustment for multiple comparisons
☐	☒	A full description of the statistical parameters including central tendency (e.g. means) or other basic estimates (e.g. regression coefficient) AND variation (e.g. standard deviation) or associated estimates of uncertainty (e.g. confidence intervals)
☐	☒	For null hypothesis testing, the test statistic (e.g. *F*, *t*, *r*) with confidence intervals, effect sizes, degrees of freedom and *P* value noted<br>*Give P values as exact values whenever suitable.*
☒	☐	For Bayesian analysis, information on the choice of priors and Markov chain Monte Carlo settings
☒	☐	For hierarchical and complex designs, identification of the appropriate level for tests and full reporting of outcomes
☒	☐	Estimates of effect sizes (e.g. Cohen's *d*, Pearson's *r*), indicating how they were calculated

Our web collection on statistics for biologists contains articles on many of the points above.

Software and code

Policy information about availability of computer code

Data collection	Western blot bands were visualized using Evolution Capt EDGE software (Ver. 18,02, M&S Instruments Inc.).<br>qPCR data was acquired on CFX Maestro (Ver. 2.3(5.3.022.1030), Bio-Rad).
Data analysis	Data were analyzed using Excel (for Mac 16.54; Microsoft).<br>ALPHA profile was analyzed using Origin (Ver. 7.5, OriginLab) or GraphPad Prism software (version 5.04).<br>Crystal structure determination was analyzed using XDS version Jan 26, 2018 for X-ray data processing, CCP4 7.0.058 including AIMLESS for X-ray data scaling, Phaser 2.8.2 for phase determination, eLBOW 1.13-2998 for ligand building, Phenix version 1.18.2 for refinement, and COOT version 0.8.9.2 for model building.<br>Statistical analyses were performed using Excel (for Mac 16.54; Microsoft) and R (version 3.5.3).<br>ChIP-seq analysis were analyzed Cutadapt (V1.9.1) for QC, FastQC (V0.10.1) for QC assessment, Bowtie (V2.1.0) for mapping, strand cross-correlation (SCC) for ChIP quality assessment, MACS2 for peak calling, and R package ChIPpeakAnno for Peak annotated.

For manuscripts utilizing custom algorithms or software that are central to the research but not yet described in published literature, software must be made available to editors and reviewers. We strongly encourage code deposition in a community repository (e.g. GitHub). See the Nature Portfolio guidelines for submitting code & software for further information.

Data

Policy information about availability of data

All manuscripts must include a data availability statement. This statement should provide the following information, where applicable:

- Accession codes, unique identifiers, or web links for publicly available datasets
- A description of any restrictions on data availability
- For clinical datasets or third party data, please ensure that the statement adheres to our policy

The raw RNA-seq and ChIP-seq reads obtained in this study have been submitted to the DDBJ Sequence Read Archive (DRA) under accession number DRA014673 (https://ddbj.nig.ac.jp/resource/sra-submission/DRA014673). The coordinates and structure factors of the G9a–RK-701 complex have been deposited in the PDB under accession code 7X73 (https://www.rcsb.org/structure/unreleased/7X73). The structure of the G9a-UNC0638 complex (PDB ID: 3RJW) is available from the following link (https://www.rcsb.org/structure/3rjw). The mass spectrometry proteomics data have been deposited in the ProteomeXchange Consortium via the PRIDE partner repository with the dataset identifier PXD033536 and 10.6019/PXD033536 (http://proteomecentral.proteomexchange.org/cgi/GetDataset?ID=PXD033536).

Human research participants

Policy information about studies involving human research participants and Sex and Gender in Research.

Reporting on sex and gender	n/a
Population characteristics	n/a
Recruitment	n/a
Ethics oversight	n/a

Note that full information on the approval of the study protocol must also be provided in the manuscript.

Field-specific reporting

Please select the one below that is the best fit for your research. If you are not sure, read the appropriate sections before making your selection.

☒ Life sciences ☐ Behavioural & social sciences ☐ Ecological, evolutionary & environmental sciences

For a reference copy of the document with all sections, see nature.com/documents/nr-reporting-summary-flat.pdf

Life sciences study design

All studies must disclose on these points even when the disclosure is negative.

Sample size	No statistical method was used to predetermine the sample size. The chosen sample size are based on the numbers used for previous publications. For all biochemical and cell biological analyses, qPCR or western blotting experiments or related investigation are repeated at least 3 or 2 times for similar results.
Data exclusions	Data were not excluded from analysis.
Replication	All experiments were repeated independently as indicated in each Figure Legend. All attempts at replication were successful.
Randomization	No formal randomization method was used.
Blinding	Investigators were not blinded to the sample identities during data collection due to none of the analyses reported involved procedures that could be influenced by investigator bias.

Reporting for specific materials, systems and methods

We require information from authors about some types of materials, experimental systems and methods used in many studies. Here, indicate whether each material, system or method listed is relevant to your study. If you are not sure if a list item applies to your research, read the appropriate section before selecting a response.

Materials & experimental systems

n/a	Involved in the study
	x Antibodies
	x Eukaryotic cell lines
x	Palaeontology and archaeology
	x Animals and other organisms
x	Clinical data
x	Dual use research of concern

Methods

n/a	Involved in the study
	x ChIP-seq
	x Flow cytometry
x	MRI-based neuroimaging

Antibodies

Antibodies used

anti-G9a (CST; #3306; 2; 1:1000 for W.B.)
anti-EHMT1 (CST; #35005; 1; 1:1000 for W.B.)
anti-α-tubulin (Abcam; ab18251; GR3240348-1; 1:5000 for W.B.)
anti-H3K27me3 (sigma; 07-449; 2506493; 1:3000 for W.B.)
anti-H3K9me2 (Abcam; ab1220; GR3228498-2; 1:3000 for W.B. and 1:100 for ChIP)
anti-H3K9ac (Active motif; 39917; 12517005; 1:100 for ChIP)
anti-H3 (Abcam; ab1791; GR3237685-2; 1:5000 for W.B.)
Goat anti-mouse IgG (H+L, Jackson Immunoresearch; 115-001-003; 1:100 for ChIP)
anti-BCL11A (Novus Biologicals; NB600-261; A2; 1:100 for ChIP)
anti-BCL11A (abcam; ab19487; GR3206086-5; 1:1000 for W.B.)
anti-ZBTB7A (Thermo Fisher Scientific; eBioscience (13E9); 2296309; 1:100 for ChIP)
anti-ZBTB7A (CST; #50565; 1; 1:1000 for W.B.)
anti-CHD3 (Novus Biologicals; NB100-60412; A2; 1:1000 for W.B. and 1:100 for ChIP)
anti-CHD4 (abcam; ab240640; GR3391298-2; 1:1000 for W.B. and 1:100 for ChIP)
anti-FLAG (Fujifilm; 01422383; 1E6; 100 ng/ml for W.B.)
anti-His (MBL; PM032; 017; 1:5000 for W.B.)
anti-mouse IgG HRP (Jackson Immunoresearch; 115-035-003; 1:20000 for W.B.)
anti-rabbit IgG HRP (Jackson Immunoresearch; 111-035-144; 1:10000 for W.B.)
FITC Mouse Anti-Human CD45 (BD Biosciences, 555482, 1:1 for flow cytometry)
APC Mouse Anti-Human CD235a (BD Biosciences, 551336, 1:20 for flow cytometry)
Human Fetal Hemoglobin APC Conjugate (Invitrogen, #MHFH05, 1:1 for flow cytometry)

Validation

anti-G9a (https://www.cellsignal.jp/products/primary-antibodies/g9a-ehmt2-c6h3-rabbit-mab/3306)
anti-EHMT1 (https://www.cellsignal.com/products/primary-antibodies/ehmt1-e6q8b-rabbit-mab/35005)
anti-α-tubulin (https://www.abcam.co.jp/alpha-tubulin-antibody-microtubule-marker-ab18251.html)
anti-H3K27me3 (https://www.merckmillipore.com/JP/ja/product/Anti-trimethyl-Histone-H3-Lys27-Antibody,MM_NF-07-449)
anti-H3K9me2 (https://www.abcam.co.jp/histone-h3-di-methyl-k9-antibody-mabcam-1220-chip-grade-ab1220.html)
anti-H3K9ac (https://www.activemotif.com/catalog/details/39917/histone-h3-acetyl-lys9-antibody-pab-2)
anti-H3 (https://www.abcam.co.jp/histone-h3-antibody-nuclear-marker-and-chip-grade-ab1791.html)
anti-mouse IgG (https://www.jacksonimmuno.com/catalog/products/115-001-003)
anti-BCL11A (https://www.novusbio.com/products/ctip1-antibody_nb600-261)
anti-BCL11A (https://www.abcam.co.jp/ctip1bcl-11a-antibody-14b5-ab19487.html)
anti-ZBTB7A (https://www.thermofisher.com/antibody/product/Pokemon-LRF-Antibody-clone-13E9-Monoclonal/14-3309-82)
anti-ZBTB7A (https://www.cellsignal.jp/products/primary-antibodies/lrf-pokemon-d7u2o-rabbit-mab/50565)
anti-CHD3 (https://www.novusbio.com/products/chd3-antibody_nb100-60412)
anti-CHD4 (https://www.abcam.co.jp/chd4-antibody-epr22953-38-chip-grade-ab240640.html)
anti-FLAG (https://labchem-wako.fujifilm.com/jp/product/detail/W01W0101-2238.html)
anti-His (https://ruo.mbl.co.jp/bio/dtl/A/?pcd=PM032)
anti-mouse IgG HRP (validated with W.B. by us and previous studies)
anti-rabbit IgG HRP (validated with W.B. by us and previous studies)
FITC Mouse Anti-Human CD45 (https://www.bdbiosciences.com/en-ca/products/reagents/flow-cytometry-reagents/research-reagents/single-color-antibodies-ruo/fitc-mouse-anti-human-cd45.555482)
APC Mouse Anti-Human CD235a (https://www.citeab.com/antibodies/2410696-551336-bd-pharmingen-apc-mouse-anti-human-cd235a)
Human Fetal Hemoglobin APC Conjugate (https://www.thermofisher.com/antibody/product/Fetal-Hemoglobin-Antibody-clone-HBF-1-Monoclonal/MHFH05)

Eukaryotic cell lines

Policy information about cell lines and Sex and Gender in Research

Cell line source(s)

HUDEP-2, HiDEP-1 and HEK293T cells were obtained from RIKEN BRC. H9c2 cells (CRL-1446) were obtained from ATCC. Human CD34+ progenitor cells derived from bone marrow or peripheral blood were obtained from Lonza. PLAT-E cells were provided from Dr. T. Kitamura of The University of Tokyo. BCL11A and ZBTB7A KO HUDEP-2 cells were provided from Dr.

	Maeda of Kyusyu University.
Authentication	Cell lines used were not authenticated.
Mycoplasma contamination	Cell lines were not tested for mycoplasma contamination.
Commonly misidentified lines (See ICLAC register)	No commonly misidentified cell line was used.

Animals and other research organisms

Policy information about studies involving animals; ARRIVE guidelines recommended for reporting animal research, and Sex and Gender in Research

Laboratory animals	As described in Methods, C57BL/6 mice (6–8 weeks old, female, six mice/group) were purchased from Tokyo Laboratory Animals Science.<br>Four-week-old female ICR mice were purchased from Charles River Breeding Laboratories (Yokohama, Japan).<br>Pharmacokinetic studies were performed using 8-week-old male Crl:CD1 ICR mice.
Wild animals	No wild animals were used.
Reporting on sex	As described in Methods, C57BL/6 mice (6–8 weeks old, female, six mice/group) were used in figure 2f-h. Pharmacokinetic studies were performed using 8-week-old male Crl:CD1 ICR mice. Mouse acute toxicity studies 4-week-old female ICR mice. In vivo micronucleus studies were used bone marrow cells obtained from the right femur of 5 male rats.
Field-collected samples	No field-collected samples ware used in this study.
Ethics oversight	As described in Methods, animal experiments were conducted in accordance with the animal experimental protocol and guidelines of the Tokyo University of Pharmacy and Life Sciences Animal Experimentation Regulations after review by the Institutional Animal Care and Use Committee (permission numbers L18-10 and L19-26) and approval by the President of the Tokyo University of Pharmacy and Life Sciences.<br>PK studies were performed in accordance with the animal experimental protocol, and procedures were approved by the Animal Care and Ethics Committee of Sekisui Medical.<br>Mouse acute toxicity assay were approved by the Institutional Committee for Animal Experiments of the Institute of Microbial Chemistry, and were performed in accordance with relevant guidelines and regulations to minimize animal suffering.

Note that full information on the approval of the study protocol must also be provided in the manuscript.

ChIP-seq

Data deposition

- [x] Confirm that both raw and final processed data have been deposited in a public database such as GEO.
- [x] Confirm that you have deposited or provided access to graph files (e.g. BED files) for the called peaks.

Data access links *May remain private before publication.*	the DDBJ Sequence Read Archive (DRA) under accession number DRA014673
Files in database submission	ChIP-DMSO, ChIP-DMSO-2, ChIP-RK, and ChIP-RK-2
Genome browser session (e.g. UCSC)	Using integrative genome viewer

Methodology

Replicates	Two replicates were performed in HUDEP-2 cells.
Sequencing depth	Sequencing was carried out using a 2x150 paired-end (PE) configuration; image analysis and base calling were conducted by the HiSeq Control Software (HCS) + OLB + GAPipeline-1.6 (Illumina) on the Illumina instrument.<br>The original image data were analyzed using CASAVA(v1.8.2) for base calling and preliminary quality analysis.
Antibodies	anti-H3K9me2 (Abcam; ab1220; GR3228498-2; 1:100 for ChIP)
Peak calling parameters	Use strand cross-correlation (SCC) for ChIP quality assessment, MACS2 for peak calling, and R package ChIPpeakAnno for Peak annotated.
Data quality	Raw data (Pass Filter Data) was processed by adapter trimming and low quality read removal using NGS quality control software Cutadapt (v1.9.1) to generate clean data for subsequent analysis. software ： Cutadapt (version 1.9.1) The process includes the following steps: (1) remove the adapter sequences (2) remove the 5 'or 3' end bases of quality scores below 20 (3) remove the reads in which 'N' is above 10% (4)remove reads that are less than 75 bp long after trimming.
Software	Cutadapt (V1.9.1) for QC, FastQC (V0.10.1) for QC assessment, Bowtie (V2.1.0) for mapping, strand cross-correlation (SCC) for ChIP

quality assessment, MACS2 for peak calling, and R package ChIPpeakAnno for Peak annotated

Flow Cytometry

Plots

Confirm that:

- [x] The axis labels state the marker and fluorochrome used (e.g. CD4-FITC).
- [x] The axis scales are clearly visible. Include numbers along axes only for bottom left plot of group (a 'group' is an analysis of identical markers).
- [x] All plots are contour plots with outliers or pseudocolor plots.
- [x] A numerical value for number of cells or percentage (with statistics) is provided.

Methodology

Sample preparation	As described in Methods, HUDEP-2 cells or human CD34+ cell-derived erythroid cells were fixed in 0.05% glutaraldehyde (Sigma) for 10 min, then washed two times with PBS containing 2% FBS (PBS/2% FBS) and permeabilized with 0.1% Triton X-100 (prepared in PBS/2% FBS; Life Technologies) for 5 min. After washing with PBS/2% FBS two times, cells were stained with allophycocyanin (APC)-conjugated HbF antibody (Invitrogen). One million cells were incubated with the HbF–APC antibody for 30 min in the dark at 4°C. Cells were washed twice with PBS/2% FBS, then suspended in 0.2 ml PBS/2% FBS and analyzed using a FACSCalibur (BD Biosciences). To assess erythroid differentiation, human CD34+ cell-derived erythroid cells were stained with FITC-conjugated CD45 antibody (BD Biosciences) and APC-conjugated CD235a antibody (BD Biosciences). Then cells were suspended in 0.2 ml PBS/2% FBS containing 1 μg/ml propidium iodide (Sigma).
Instrument	BD FACSCalibur was used for analysis.
Software	FlowJo software v10.7.1(BD Biosciences)
Cell population abundance	Sorted fractions were not used for analysis
Gating strategy	Cell debris was excluded by FSC/SSC gates and for CD45/CD235a phenotype analysis, Propidium Iodide (PI) were used for dead cell exclusion.

- [x] Tick this box to confirm that a figure exemplifying the gating strategy is provided in the Supplementary Information.
